# An Easy‐to‐Implement Toolkit to Create Versatile and High‐Performance HASEL Actuators for Untethered Soft Robots

**DOI:** 10.1002/advs.201900178

**Published:** 2019-06-11

**Authors:** Shane K. Mitchell, Xingrui Wang, Eric Acome, Trent Martin, Khoi Ly, Nicholas Kellaris, Vidyacharan Gopaluni Venkata, Christoph Keplinger

**Affiliations:** ^1^ Department of Mechanical Engineering University of Colorado Boulder Boulder CO 80309 USA; ^2^ School of Physics Science and Engineering Tongji University Shanghai 200092 China; ^3^ Department of Electrical Computer & Energy Engineering University of Colorado Boulder Boulder CO 80309 USA; ^4^ Materials Science and Engineering Program University of Colorado Boulder Boulder CO 80309 USA

**Keywords:** artificial muscles, bioinspired robots, electroactive polymers, HASEL actuators, soft robotics

## Abstract

For soft robots to have ubiquitous adoption in practical applications they require soft actuators that provide well‐rounded actuation performance that parallels natural muscle while being inexpensive and easily fabricated. This manuscript introduces a toolkit to rapidly prototype, manufacture, test, and power various designs of hydraulically amplified self‐healing electrostatic (HASEL) actuators with muscle‐like performance that achieve all three basic modes of actuation (expansion, contraction, and rotation). This toolkit utilizes easy‐to‐implement methods, inexpensive fabrication tools, commodity materials, and off‐the‐shelf high‐voltage electronics thereby enabling a wide audience to explore HASEL technology. Remarkably, the actuators created from this easy‐to‐implement toolkit achieve linear strains exceeding 100%, a specific power greater than 150 W kg^−1^, and ≈20% strain at frequencies above 100 Hz. This combination of large strain, extreme speed, and high specific power yields soft actuators that jump without power‐amplifying mechanisms. Additionally, an efficient fabrication technique is introduced for modular designs of HASEL actuators, which is used to develop soft robotic devices driven by portable electronics. Inspired by the versatility of elephant trunks, the above capabilities are combined to create an untethered continuum robot for grasping and manipulating delicate objects, highlighting the wide potential of the introduced methods for soft robots with increasing sophistication.

## 1 Introduction

The field of soft robotics challenges the conventional definition of a robot by substituting motors, gears, and metal with more compliant and multifunctional components inspired by those found in biological systems.^1^, ^2^, ^3^ Soft robots offer an intrinsic adaptability and dexterity that is well‐suited for safe operation near humans, opening applications in wearable, surgical and collaborative robotics.^2^, ^4^, ^5^, ^6^, ^7^ Moreover, these lightweight and versatile machines can find utility in aerospace and marine engineering, industrial processing and automation, and active camouflaging.^3^, ^8^, ^9^, ^10^

Soft actuators that mimic the universal performance of natural muscle^11^, ^12^ are critical components for creating the next generation of soft robots that achieve levels of functionality seen only in biological systems. Two widespread types of soft actuators are soft fluidic actuators and dielectric elastomer actuators. Soft fluidic actuators utilize a pressurized fluid (usually air or water) to drive shape change of a deformable architecture (usually based on flexible or stretchable polymers). These devices offer diverse modes of actuation,^2^, ^13^, ^14^, ^15^, ^16^ but typically require a tethered connection to sources of pressurized fluid, which limits speed, efficiency, and portability. Dielectric elastomer actuators (DEAs) are stretchable capacitors that employ electrostatic activation to achieve large actuation strain, high‐speed operation, and self‐sensing capabilities enabling closed‐loop control.^17^, ^18^, ^19^, ^20^, ^21^ However, DEAs are vulnerable to failure by dielectric breakdown when scaled to large activation areas^22^ and require stretchable materials for both electrodes and dielectric layers, which restricts material selection and design freedom.

Recent work by Acome et al.^23^ and Kellaris et al.^24^ introduced hydraulically amplified self‐healing electrostatic (HASEL) actuators that blend the versatility of soft fluidic actuators with the muscle‐like performance of DEAs, while simultaneously addressing key drawbacks. HASEL actuators use electric fields to locally displace liquid dielectrics enclosed in soft hydraulic architectures, which mitigates losses in speed and efficiency associated with transporting fluid through a system of channels (typically seen in soft fluidic actuators). In contrast to DEAs, the use of a liquid dielectric allows HASELs to electrically self‐heal from dielectric breakdown to improve reliability.^23^ Importantly, the electrohydraulic driving mechanism of HASEL actuators is compatible with a wide range of stretchable and also flexible yet inextensible materials that are not available to DEAs (particularly for electrodes); HASELs have been built using compliant ionic conductors but also flexible metallized layers as electrodes.^24^ While initial prototypes of HASELs^23^, ^24^ provided muscle‐mimetic actuation characteristics, achieving such performance required very high voltages (HVs) (≈20 kV), which severely restricted the options for commercially available miniature high‐voltage amplifiers for portable driving electronics. Additionally, these initial prototypes were based on fabrication techniques that utilized a mold to cast elastomers or a metal die to act as a heat‐sealing press, both of which required time‐consuming machining steps to be adjusted to different geometries and designs.

Here, we introduce a versatile toolkit to rapidly prototype a wide range of designs of HASEL actuators that achieve all three basic modes of actuation while incorporating electrostatic zipping mechanisms to reduce operating voltages. Relying only on a programmable three‐axis computer numerically controlled (CNC) machine and commercially available materials, the presented approach provides a simple, accessible, and effective method to design and construct HASEL actuators with tailorable actuation characteristics; we show designs that can linearly expand or contract, as well as curl or twist. Notably, the HASELs fabricated with this easy‐to‐implement method achieved remarkable actuation performance with actuation strains up to 118%, strain rates of about 7500% s^−1^, a roll‐off frequency of 126 Hz, and a peak specific power of 156 W kg^−1^. For untethered operation of the actuators, we also developed portable high‐voltage driving electronics using off‐the‐shelf miniature components. Further, we introduce a modular design of robotic devices based on stacks of HASELs and demonstrate an efficient fabrication process to rapidly construct these stacks through a folding technique. Finally, we utilize modular actuators to build an untethered soft continuum robot capable of grasping and manipulating delicate objects.

## 2 Results

### 2.1 Electrostatic Zipping Mechanisms in HASEL Actuators

**Figure 1:**
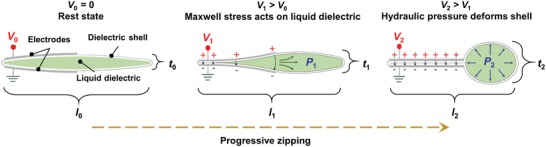
Generalized principle of zipping‐mode actuation in HASEL actuators. As voltage is increased from *V*_0_ to *V*_2_, the electrodes progressively zip together beginning at the left tapered edge. This electrostatic zipping results in a gradual change in thickness, *t*, and length, *l*, of the soft hydraulic structure.

The HASEL actuators developed here incorporate electrostatic zipping mechanisms to achieve a continuous actuation response without dramatic pull‐in instabilities while reducing the operating voltages required for large deformations. Electrostatic zipping is a well‐known phenomenon in micro‐electromechanical systems (MEMS) and DEAs^25^, ^26^, ^27^ and has more recently been demonstrated in electrohydaulic origami actuators.^28^ **Figure** **1** shows the generalized principle of zipping‐mode actuation in HASEL actuators. A thin, flexible, and inextensible dielectric shell encapsulates a liquid dielectric and electrodes are placed on either side of a tapered edge of the shell. At *V*_0_ = 0, the actuator is at its rest state with initial thickness, *t*_0_, and initial length, *l*_0_. Increasing voltage from *V*_0_ to *V*_1_, causes a gradient in electric field, with the highest field located at the tapered edge. This field generates an electrostatic Maxwell stress acting on the dielectric layers, governed by σ ∝ *εE* ^2^, where ε is the permittivity of the dielectric layers and *E* is the electric field.^29^ The electrodes begin to “zip” together starting at the tapered edge. This process results in an increase of the hydraulic pressure within the shell, *P*_1_, which causes part of the soft structure to increase in thickness to *t*_1_ and decrease in length to *l*_1_. As voltage is further increased to *V*_2_ the electrodes progressively zip together until all the liquid dielectric is displaced into the region of the shell that is not covered by electrodes. In this state, the hydraulic pressure within the shell is at *P*_2_ > *P*_1_, and the soft structure deforms to a maximum thickness, *t*_2_, and a minimum length, *l*_2_.

### 2.2 Fabrication Process to Rapidly Prototype Various Types of HASEL Actuators

**Figure 2:**
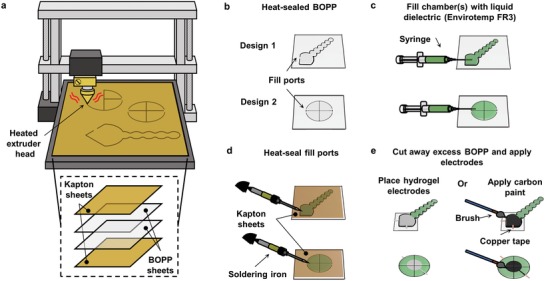
Fabrication process to rapidly prototype various types of HASEL actuators. a) An illustration of the three‐axis CNC heat sealing machine used to bond thermoplastics for the dielectric shell. b) Two exemplary patterns of heat seals used in HASEL actuators. Small gaps in the seal were incorporated to c) fill the shell with liquid dielectric using a needle and syringe. d) Once the shell was filled with liquid dielectric, the fill ports were sealed with a soldering iron. e) The excess BOPP was cut away and electrodes were applied to either side of the actuator.

To incorporate zipping mechanisms in various types of HASEL actuators, we aimed to identify a material system and fabrication technique which would allow us to rapidly prototype various geometries of the dielectric shell. A three‐axis CNC machine can be used to heat seal thin layers of thermoplastics into pouches of various shapes and sizes.^30^, ^31^, ^32^ Here, we repurposed an inexpensive 3D printer into a three‐axis CNC heat sealing machine (**Figure** **2**a) and used it to thermally bond sheets of biaxially oriented polypropylene (BOPP). BOPP was chosen since it is an inexpensive thermoplastic with excellent dielectric strength (≈700 V µm^−1^) and tensile strength (≈300 N mm^−2^),^24^ but many other types of thermoplastics are compatible with this heat sealing method. A biodegradable vegetable‐based transformer oil (Envirotemp FR3, Cargill) was chosen as the liquid dielectric because of its favorable dielectric properties, nontoxicity, and low viscosity. Similar to the dielectric shell, many other types of liquid dielectrics with specific desired properties are also compatible with this fabrication technique. In contrast to DEAs, HASELs do not require stretchable electrodes; only flexible conductors are necessary,^24^ allowing the use of many different types of conductors for electrodes. In this work, we used ionically conductive polyacrylamide (PAM) hydrogels to enable transparent designs of HASELs^23^, ^24^ (Figure S1, Supporting Information), as well as off‐the‐shelf and inexpensive carbon‐based conductive paint that was easily applied with a paint brush. Figure 2 illustrates the fabrication process using the specific materials described above. Movie S1 (Supporting Information) demonstrates the fabrication process from start to finish.

### 2.3 Bioinspired Designs of HASEL Actuators

**Figure 3:**
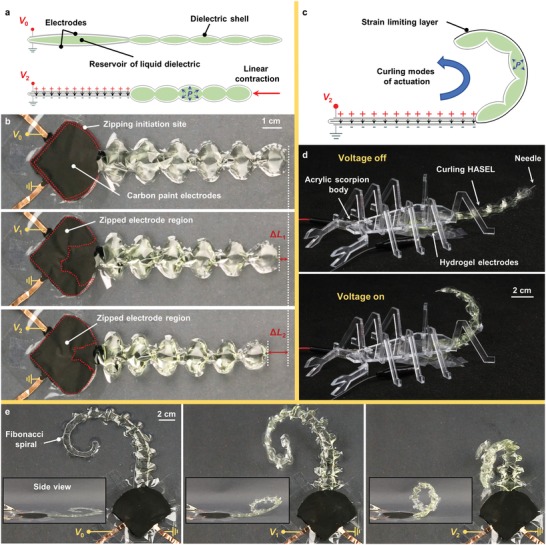
Rapid prototyping of HASELs for bioinspired designs that contract, curl, and twist. a) A schematic of a contracting HASEL actuator. b) A prototype of a contracting actuator. The zipping mechanism allowed for variable contraction upon application of voltage. c) A strain limiting layer applied to one side of the contracting actuator forces the device to curl upon application of voltage. d) A curling actuator was used to mimic the high‐speed strike of a scorpion tail. e) The corrugated shape of the heat seal was designed as a Fibonacci spiral. As voltage was increased from *V*_0_ to *V*_2_, the spiral curled upward while simultaneously generating a twisting motion.

The easy‐to‐implement fabrication technique presented in this work allowed us to easily explore new geometries of HASEL actuators. Inspired by different designs of soft pneumatic actuators made from thin film polymers,^33^, ^34^ we prototyped HASEL actuators that contract, curl, and twist upon activation. **Figure** **3** shows the basic design and operating principle of these actuators. The contracting actuators feature electrodes which cover a reservoir of liquid dielectric that leads into a corrugated pathway, Figure 3a. Upon activation the electrodes zip together forcing the liquid dielectric into the corrugated pathway. Since the dielectric shell is inextensible, the increased pressure within the corrugations causes the radius of curvature of each corrugation to decrease, resulting in an overall contraction of the soft hydraulic structure. Figure S2 (Supporting Information) shows a few design iterations of these contracting actuators, and the final version is presented in Figure 3b. Carbon paint electrodes were painted to the perimeter of the heat seal which constituted the reservoir of liquid dielectric, thereby creating a zipping initiation site along this perimeter. When voltage *V*_1_ was applied, the electrodes began to zip together, resulting in a length change of the actuator, Δ*L*_1_. The applied voltage was further increased to a maximum value of *V*_2_, which caused a maximum length change of Δ*L*_2_.

The contracting actuators described above can be modified to curl by adding a strain limiting layer to one side of the actuator, Figure 3c. This layer constrains one side of the shell, resulting in a preferential deformation of the unconstrained side. This deformation leads to a differential contraction and overall bending movement, similar to pneumatic bending actuators.^35^ The rapid actuation of these curling HASELs resembled the high‐speed strike of a scorpion tail, Figure 3d and Movie S2 (Supporting Information). Figure S3 (Supporting Information) shows that the peak velocity of the tip of the curling actuator is 1.26 m s^−1^, which is nearly identical to the maximum velocity of a tail strike from the scorpion species *Leiurus quinquestriatus*.^36^ Additionally, the curling actuator achieved a maximum angular velocity of 1987° s^−1^ and a blocked tip force of 550 mN (Figure S3, Supporting Information). We also demonstrated independent control of three curling HASEL actuators using a three‐channel HV power supply, Movie S2 (Supporting Information).

Furthermore, the shape of the corrugated pathway can be modified for added functionality. We prototyped the heat seal of the corrugated pathway in the shape of a Fibonacci spiral, and attached a strain limiting layer to one side of the actuator, Figure 3e. Upon activation, the spiral shape curled upward while simultaneously generating a torsional deformation, Movie S3 (Supporting Information), which resembles the contorting motion of tendrils found in certain plant species.

### 2.4 Improving the Performance of Donut HASEL Actuators through Rapid Prototyping

Donut HASEL actuators presented by Acome et al.^23^ showed promise as high speed and efficient linear actuators with tailorable stress‐strain curves through electrohydraulic coupling. However, these actuators required voltages above 15 kV to achieve strains of about 40% under loads of ≈1 N. These voltages were necessary to produce high electric fields across the relatively thick (≈1 mm) silicone elastomers used as the dielectric shell.

**Figure 4:**
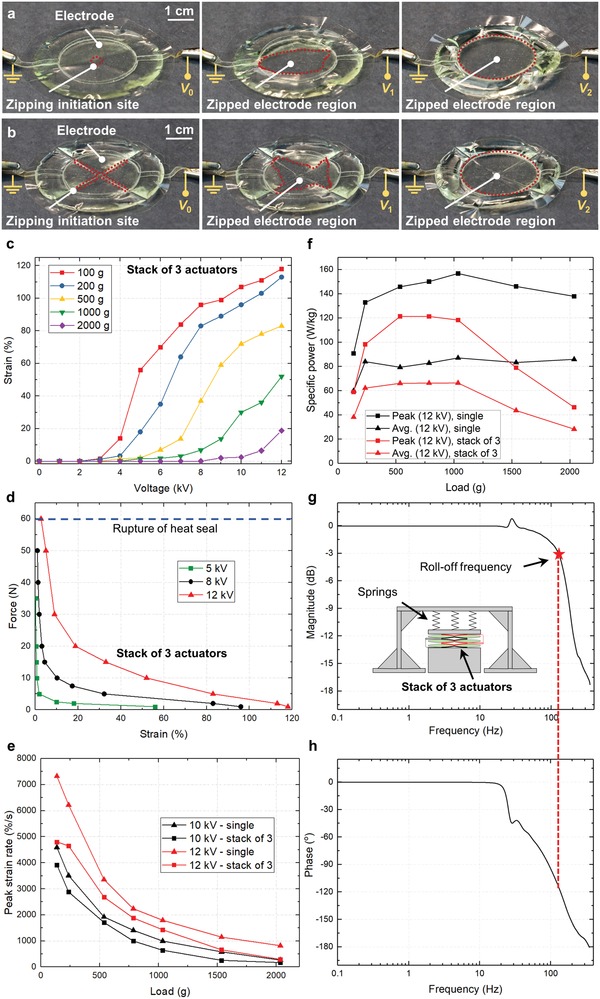
Design and characterization of stackable quadrant donut HASELs for large strain, high‐power, and high‐speed linear actuation. a) The first design iteration used a central heat seal (“dimple”) in a donut HASEL actuator to initiate zipping‐mode actuation from this central site. As voltage was gradually increased from *V*_0_ = 0 kV to *V*_1_ = 2 kV to *V*_2_ = 5 kV, the electrodes progressively zipped together without pull‐in instabilities. b) To ensure a more homogeneous distribution of liquid dielectric during actuation, the shell of a donut HASEL actuator was divided into four equal quadrants using an “X”‐shaped heat seal. This “X” acts as the zipping initiation site, and the electrodes progressively zip together as voltage is increased from *V*_0_ to *V*_2_. c–h) Performance metrics for a stack of three quadrant donut HASELs; panels (e) and (f) additionally show metrics for a single quadrant donut HASEL actuator. g,h) Bode plot for the stack of three actuators, where 0 dB corresponds to 25% strain. The actuators achieved muscle‐like strains (≈20%) beyond 100 Hz. A spring platform was used to provide a restoring force during characterization of frequency response.

Using the CNC heat sealing machine and thin‐film BOPP (18 µm thick) as the dielectric shell, we prototyped donut HASEL actuators which featured electrostatic zipping mechanisms. To better visualize the zipping mechanisms, transparent ionic conductors were used for electrodes; **Figure** **4**a shows a basic design for a zipping donut actuator wherein electrodes are placed concentric to a small circular heat seal that was also concentric with the outer circumference of the actuator. The central heat seal resembled a dimple and acted as a zipping initiation site; when voltage was applied, the electrodes began to zip radially outward from the central dimple of the actuator. As the electrodes zipped together, liquid dielectric was forced away from the central dimple to the outer regions of the shell, causing the actuator to transition from a disc shape to a toroidal or donut shape. A schematic of a dimpled donut HASEL actuator is shown in Figure S4 (Supporting Information).

Dimpled donut HASELs showed a continuous response to input voltage and began to actuate at voltages as low as 2 kV. However, it was observed that when a few of these actuators were stacked on top of each other and placed under a mechanical load, the liquid dielectric in adjacent actuators was distributed unevenly in the outer regions of the shells, inhibiting each actuator in the stack from taking a toroidal shape. This uneven distribution limited the linear expansion of the stack to values lower than expected from extrapolation of the strain achieved by a single actuator.

Figure 4b shows a donut HASEL actuator with a dielectric shell that was segmented into quadrants, each filled with the same volume of liquid dielectric. This segmentation resulted in a more homogenous distribution of the liquid dielectric during actuation under load, while also providing a larger zipping initiation site compared to the dimpled donut actuators. Figure S5 (Supporting Information) compares dimpled donuts to quadrant donut HASELs under a 500 g load. Figure S5a (Supporting Information) shows that one dimpled donut achieved a larger actuation strain (11% at 4 kV and 56% at 10 kV) as compared to one quadrant donut (2% at 4 kV and 41% at 10 kV). However, a stack of three dimpled donuts generated an actuation strain of only 20% at 10 kV, whereas a stack of three quadrant donuts achieved 72% at 10 kV, Figure S5b (Supporting Information).

The actuation characteristics of a stack of three quadrant donut HASELs are shown in Figure 4c–h, where Figure 4e,f provide additional information on the performance of single quadrant donut actuators. Under a 1 N load, the stack of actuators achieved a linear strain of 15% at 4 kV and a maximum strain of 118% at 12 kV, Figure 4c. The blocked force for this stack of actuators was above 60 N and was ultimately limited by the strength of the heat seals, Figure 4d. The large zipping initiation site afforded by the segmented shell promoted high‐speed actuation, and Figure 4e shows the stack achieved a strain rate of 4800% s^−1^ at 12 kV under a 1 N load, and nearly 300% s^−1^ under a 20 N load. Furthermore, the stack of three actuators demonstrated a peak specific power of 121 W kg^−1^ (Figure 4f and Figure S6, Supporting Information). Using the specific test setup shown in Figure 4g, which consisted of three springs and a rigid acrylic platform to provide a restoring force, we measured a flat frequency response at 25% strain until roll‐off at 126 Hz (Figure 4g,h). The small peak seen in the frequency response (≈30 Hz) (Figure 4g) was attributed to a resonance in the test setup. Using a test setup with a different restoring force would affect these results; a lower restoring force would allow the actuators to achieve a larger stroke and generate more inertia, and therefore the roll‐off frequency would decrease. Conversely, if a larger restoring force was used, the actuators would achieve less strain, but would roll‐off at higher frequencies.

The full‐cycle electromechanical efficiency of the actuators was also measured. The experimental setup is shown in Figure S7 (Supporting Information) and was similar to the technique used by Acome et al.^23^ The results are summarized in Figure S8 (Supporting Information), which show the efficiency of the actuators as a function of the number of actuators within a stack. We observed that a single quadrant donut HASEL achieves a full‐cycle efficiency of 19.4%, a stack of three quadrant donut HASELs was 15.6% efficient, and a stack of 11 actuators yielded an efficiency of 13.6%. Additional details on experimental methods and calculations for performance metrics can be found in the Experimental Section and the Supporting Information.

### 2.5 Modular Units of Quadrant Donut HASELs

**Figure 5:**
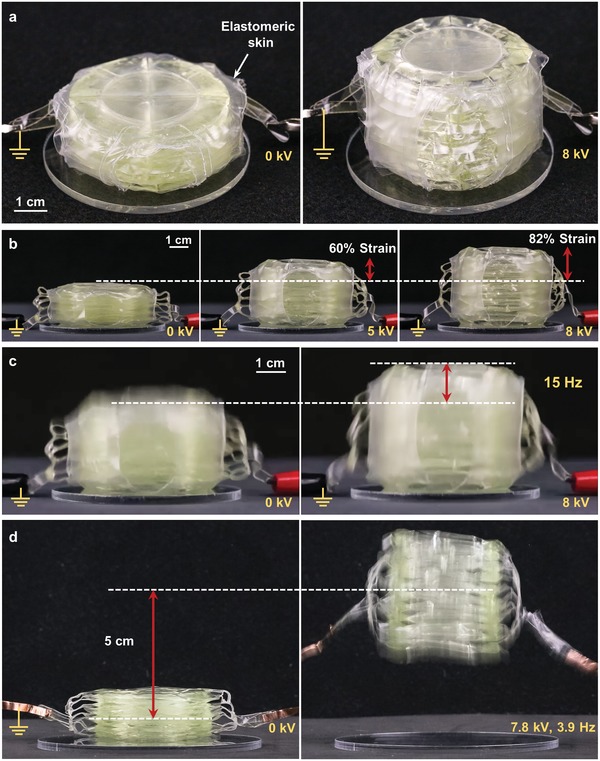
Demonstration of performance of stacked quadrant donut HASELs. a) An aerial view of a modular unit of actuators, defined as a stack of 11 quadrant donut HASELs. b) These modular units demonstrate a smooth actuation response to input voltage, with strains of 60% at 5 kV and 82% at 8 kV. c) The stacked actuators maintain large strains (>40%) at high frequencies (15 Hz). d) High specific power enables actuators that jump without additional power‐amplifying mechanisms.

We used quadrant donut HASELs to create modular units of stacked actuators (**Figure** **5**) that achieve large actuation strokes. Each unit consisted of 11 quadrant donut HASELs and a thin elastomeric “skin” made from Ecoflex 00–10 wrapped around the stack to provide an elastic restoring force. The large actuation strokes are possible without a dramatic change in the diameter of the device due to the fact that the overall volume occupied by the stack increases during actuation (the electrodes of adjacent actuators move apart from each other thereby creating voids between the electrodes). Figure S9 (Supporting Information) details the construction process for these modular units. Movie S4 (Supporting Information) demonstrates actuation of a modular unit when powered by a Trek Model 50/12 high‐voltage amplifier. The voltage was applied using custom waveforms shown in Figure S10 (Supporting Information) which reversed the polarity of the applied voltage after each actuation cycle. Reversing polarity mitigated charge retention within the actuator during continued cycling. Without reversing polarity, it was observed that charge retention would prevent the actuator from fully returning to its initial position. Figure 5a shows an aerial view of a modular unit actuating at 8 kV, while Figure 5b shows a side view of the unit at three different voltages. Additionally, large actuation strains (≈40%) were observed at driving frequencies of 15 Hz, Figure 5c. Moreover, the segmented shell of each actuator in the modular unit confines the liquid dielectric to discrete regions of each actuator and the elastomeric wrap provides a restoring force to the module, enabling the unit to effectively operate in various orientations regardless of the effects of gravity, as exemplified by Movie S4 (Supporting Information). Additionally, the strain as a function of voltage for a modular unit of quadrant donut HASELs was measured at three different orientations (upright, sideways, and upside‐down). Figure S11 (Supporting Information) shows that the strain of one modular unit is largely unaffected by the orientation of the actuator (more details in the Supporting Information).

The low weight of the modular units (≈26 g) combined with their high‐speed actuation characteristics and large strokes allowed them to jump without relying on external power amplifying mechanisms (Figure 5d and Movie S5, Supporting Information). When a 7.8 kV, 3.9 Hz driving signal was applied, the stack jumped off the ground repeatedly with its center of gravity reaching a maximum height of ≈5 cm. The elastomeric skin was removed from the modular unit in this case as it adhered to surfaces preventing the actuator from fully lifting off the ground.

### 2.6 Portable High‐Voltage Electronics for Untethered Operation of HASEL Devices

**Figure 6:**
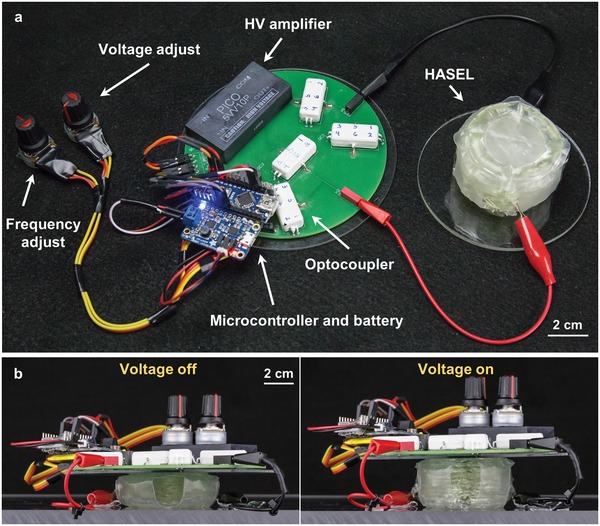
Compact high‐voltage electronics for untethered operation of HASEL actuators. a) A proof‐of‐concept power supply was constructed using only off‐the‐shelf components and was shown to operate a modular unit of actuators. b) The modular unit was operated at 8 kV and could easily lift its entire power supply including the battery.

DEAs driven by miniature high‐voltage electronics have been shown to effectively power untethered soft robots.^37^, ^38^, ^39^, ^40^ Similar to DEAs, the electrostatic driving mechanism and high specific power of HASEL actuators enables the development of compact and portable HV electronics for untethered operation of HASEL‐driven soft robots. **Figure** **6**a and Movie S6 (Supporting Information) show a modular unit of HASEL actuators powered by a portable HV electronics package. This circuit (Figure S12, Supporting Information) was based on the open source power supply developed by PetaPicoVoltron^41^ which has been utilized to drive DEAs for applications in soft robotic grippers and active substrates.^42^, ^43^ The circuit developed here was modified for higher voltage and power output; a 5 W high‐voltage amplifier was used to generate a proportional output voltage of 0–10 kV from a 0–5 V input. Optocouplers were used as high‐speed, HV switches to charge and discharge the actuators, allowing a maximum charging and discharging current of 0.3 mA. Therefore, the maximum continuous power draw by the actuators was restricted to 3 W. The optocouplers were oriented in an H‐bridge configuration to reverse the polarity of the voltage applied to the actuators. A 3.7 V, 500 mAh lithium polymer battery with a 5 V power booster were used to power the circuit, while a microcontroller was used to switch the optocouplers and modulate the voltage and frequency input to the actuator. This proof‐of‐concept electronics package fit in the palm of a hand and weighed ≈100 g. Figure 6b and Movie S6 (Supporting Information) show that a modular unit easily lifted its entire driving electronics and battery, operating completely untethered from any additional energy source. More details on powering the actuators with this HV power supply can be found in the Supporting Information.

### 2.7 An Untethered Continuum Robot Based on Modular Units of HASEL Actuators

**Figure 7:**
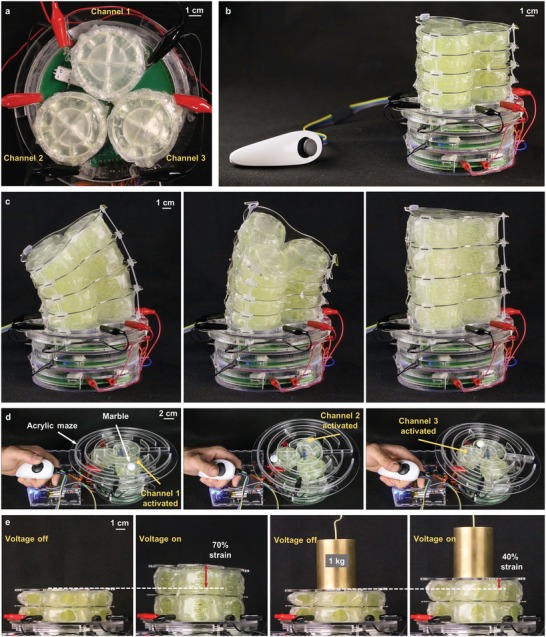
Untethered continuum robot based on modular units of HASEL actuators. a) Three modular units of HASEL actuators were oriented in a triad configuration. Each unit was connected to one channel of the custom power supply and activated with a voltage ranging from 0 to 8 kV. b) The continuum robot consisted of three columns each comprised of four modular units electrically connected in parallel. Each column was independently controlled by a joystick. c) Independent control of each column caused the robot to bend in various directions following the movement of the joystick, while activating all columns simultaneously caused the robot to linearly expand. d) An interactive game was created by placing an acrylic maze on top of three modular units of HASELs. A player navigated a marble through the maze using the joystick. e) Three columns each consisting of two modular units demonstrated 70% linear strain under no load and 40% under 1 kg.

Moving toward untethered soft robots with increasing complexity, we developed a three‐channel HV power supply for independent control of multiple modular units of HASEL actuators, **Figure** **7** and Figure S13a (Supporting Information). Three of the HV power supplies shown in Figure 6 shared a common ground and interfaced with a microcontroller which mapped analog input signals from a modified video game controller to the input of each HV amplifier, Figure S13b (Supporting Information). Therefore, by manipulating the position of the joystick on the controller, each power supply was activated independently. The output of each channel was connected to a modular unit of HASELs, and the three units were oriented in a triad configuration as seen in Figure 7a. The modular units could be stacked on top of each other and connected in parallel to further scale up the actuation stroke, Figure S14 (Supporting Information). We used three columns each consisting of four modular units to construct an untethered continuum robot capable of three‐dimensional mobility, Figure 7b. Each layer of modular units was separated by a thin acrylic spacer to provide mechanical stability to the system, and each spacer was anchored to the plate above and below it with either elastic bands made from Ecoflex 00–30 or fishing line which was used to limit the strain of each unit to 100%, Figure S15 (Supporting Information). These anchors prevented the robot from toppling over as it bent in various directions. This soft robot could bend following the movement of the joystick or linearly expand by activating all modular units simultaneously via the press of a button embedded in the controller, Figure 7c and Movie S7 (Supporting Information). Movie S7 (Supporting Information) also shows that three columns each consisting of seven modular units could be used with the three‐channel power supply to achieve bending angles of nearly 90°.

With a maximum power output of 3 W from each channel, actuation speed was limited when scaling up the number of modular units in each column as the overall value of the capacitive load increased with more modular units. If high speed operation of stacks consisting of many modular units is desired, a high‐voltage amplifier with a higher power output would yield a proportional increase in the response speed of the device. To demonstrate high‐speed operation of HASEL actuators using the portable three‐channel power supply developed here, we reduced the total capacitive load of each channel to only one modular unit and used the resulting device to precisely control the position of a marble moving through an acrylic maze, Figure 7d and Movie S8 (Supporting Information). Additionally, three columns each consisting of two modular units were shown to achieve linear strains of 70% under no load and were strong enough to achieve 40% strain under a 1 kg weight, Figure 7e and Movie S9 (Supporting Information).

### 2.8 Toward an Untethered Soft Robot for Grasping and Manipulation: Terry the Trunk

**Figure 8:**
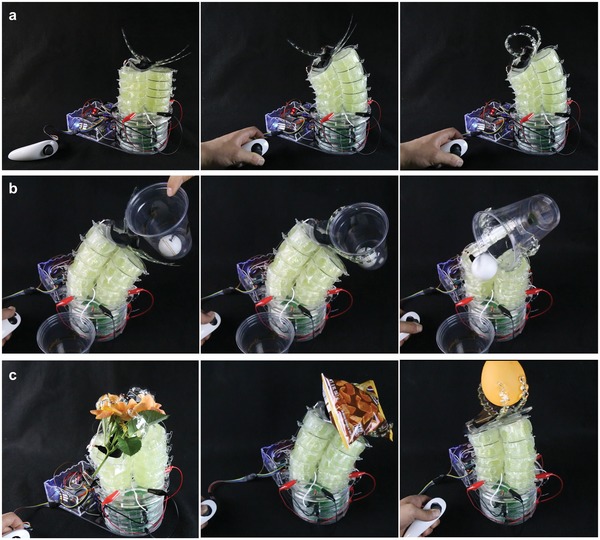
Toward an untethered soft robot for grasping and manipulation: Terry the trunk. a) Terry bent in one direction and flexed its end effector. b,c) Terry grasped and manipulated a variety of delicate objects such as a plastic cup, a flower, a bag of chips, and a balloon.

The designs of soft robots are often inspired by the rich functionality and high adaptability of continuum actuators found in nature (octopus arms, elephant trunks, tentacles and tongues, etc.).^44^, ^45^, ^46^, ^47^ Aiming to create an untethered, electrically powered soft robot based on HASEL actuators for grasping and manipulation, we combined several capabilities described in the previous sections, **Figure** **8**. We used three columns each consisting of five modular units of HASELs as a continuum actuator, while our fabrication technique allowed us to prototype a soft gripper based on curling HASEL actuators for the end effector, Figure S16 (Supporting Information). A four‐channel power supply based on the electronics described in Figure 6 and Figure 7 was used to control the three‐dimensional motion of the end effector as well as opening and closing of the gripper, Figure 8a. The HV transmission lines for the gripper were routed through the gap in the middle of the three columns, and the gripper was activated via a push button embedded in the joystick controller. This soft robot, named Terry the trunk, was adept at grasping and repositioning objects handed to it by a person, Figure 8b,c and Movie S10 (Supporting Information).

### 2.9 Folding HASEL Actuators to Create Modular Units of Actuators

The modular units of actuators shown thus far offer high‐performance actuation characteristics, but rapidly fabricating many of these devices is challenging using the techniques presented in Figure S9 (Supporting Information). In this section, we introduce an alternative method that allows the rapid production of modular units of stacked HASEL actuators through use of a folding design. This method offers a few key characteristics that make it ideal for rapidly manufacturing large quantities of modular units, but at the same time the devices constructed from this method differ from the modular units shown in Figure 5 in terms of electrical properties and robustness. These differences will be discussed in detail at the end of this section.

**Figure 9:**
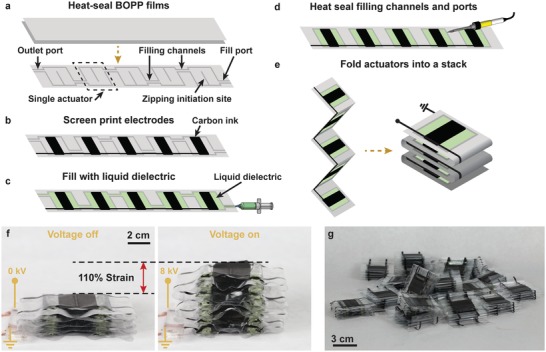
Fabrication method to rapidly produce modular units of foldable HASEL actuators. a) A heat‐seal pattern defined multiple individual HASEL actuators on a single strip of BOPP films. b) Carbon ink electrodes were screen‐printed onto the heat‐sealed films. The same pattern was applied to the bottom side of the films (not shown here). c) Liquid dielectric was inserted into all individual actuators simultaneously through the fill port and any air bubbles were expelled through the outlet port. d) Once the liquid dielectric was evenly distributed among the individual actuators, the filling channels between actuators were sealed with a soldering iron. e) The completed strip of actuators was folded into a stack. f) A folded stack of eight HASEL actuators is shown to expand under an applied voltage of 8 kV reaching a strain of 110%. g) This fabrication method made it possible to rapidly produce many modular units of HASEL actuators.

**Figure** **9** details a method to rapidly manufacture modular units of HASEL actuators through a folding technique. In this method, the dielectric shell for each actuator in the unit is heat sealed in a single step, and then the electrodes for each actuator are screen‐printed onto the dielectric shell as one continuous strip (Figure S17, Supporting Information). Next, all the actuators in the modular unit are filled with liquid dielectric simultaneously through one fill port, and the liquid is evenly distributed throughout all the actuators by placing a 2 kg rigid plate on top of the strip of actuators. Then, the rigid plate is removed and the filling channels between actuators are heat sealed using a soldering iron. Finally, stacking the actuators into a modular unit is accomplished by folding the actuators in a zigzag pattern; related folding techniques have been successfully used in the field of DEAs.^48^ The mechanical connections between adjacent actuators are provided by double‐sided tape (3M, 924 Transfer Tape). The number of actuators in a single stack is limited by the size of the heat‐sealing equipment and screen for depositing the electrodes. This method increases the production rate of one modular unit from ≈3 h (using the technique described in Figure S9, Supporting Information) to ≈30 min.

A force‐strain plot for the folded modular unit seen in Figure 9 is displayed in Figure S18a (Supporting Information) and shows that the actuator achieves a maximum strain of 110% and a maximum force of 33 N with an applied voltage of 8 kV. Figure S18b (Supporting Information) shows the full‐cycle electromechanical efficiency of the foldable HASEL actuators as a function of the number of actuators within the stack. One foldable HASEL demonstrates an efficiency of 31.2%, whereas a stack of eight actuators achieves 15.4%. Importantly, this folding approach (and therefore a modular unit) is not limited to the design seen in Figure 9 and can be applied to quadrant donut HASELs or alternative geometries such as Peano‐HASEL actuators.^24^

The modular units shown in Figures 5 and 9 exhibit key differences, besides their underlying fabrication method. First, the electrodes of adjacent actuators of the modular units in Figure 5 are connected in parallel, whereas the electrodes of the actuators in the modular units in Figure 9 are connected in series. Overall, electrodes connected in series have higher resistance than the same electrodes connected in parallel, and therefore, influence the electrical response speed (RC time) of the modular unit. Moreover, when modular units of actuators are created using the folding method, if one actuator in the module breaks, the entire module must be replaced. On the other hand, if one actuator breaks in the modular units shown in Figure 5, the broken actuator can be removed and replaced to restore functionality of the modular unit.

## 3 Conclusion

The high‐performance actuators created from the easy‐to‐implement fabrication technique presented in this work offer tunable actuation modes; they can be designed to expand, contract, rotate, or any combination of the three. These characteristics are achieved using only commodity materials (≈$0.10 in materials per actuator). However, electrical self‐healing is still unreliable using the discussed material system, as dielectric breakdown events can melt small holes in the thin thermoplastic sheets of BOPP allowing the liquid dielectric to leak from the actuator.^24^ Nevertheless, the largest electric field used to achieve the reported actuation performance is ≈333 V µm^−1^, which is well below the maximum dielectric strength of BOPP (≈700 V µm^−1^) allowing for reliable actuation without dielectric breakdown.

As a new actuator technology, HASELs require fundamental studies to guide material selection,^49^ elucidate scaling laws, and determine methods to circumvent charge retention in the actuator. Currently, charge retention within the BOPP‐based HASEL actuators necessitates reversing the polarity of voltage during subsequent actuation cycles which increases the complexity and size of the driving electronics.

Although the voltage used for actuation in this work is substantially lower than reported values by Acome et al.,^23^ it still is in the kilovolts range; thus, electrical safety needs to be considered when operating HASELs in close proximity to humans. However, it is important to note that the actuators and high‐voltage power supply developed here do not present a safety concern, because the maximum capacitance of the actuators and the charging/discharging currents are low (on the order of 10 nF and < 1 mA, respectively), while the equivalent series resistance of the electrodes is high (>1 kΩ).^50^ To further reduce the risk of accidental electrostatic discharge, the operating voltages of HASEL actuators can be decreased by using thinner solid and liquid dielectric layers and by increasing the electrical permittivity of these layers.

Untethered operation of soft robots is critical for applications like wearable and field robotics^51^ where these machines must operate without being constrained by their power sources.^52^ While the power supply developed in this work was shown to effectively drive untethered modular units of HASEL actuators through a user input, a closed‐loop controller is necessary for autonomous operation of these devices. This controller could rely on the self‐sensing mechanism inherent to HASEL actuators.^23^, ^24^, ^53^ Additionally, the use of high‐voltage power supplies, switches, and soft logic developed for DEAs^54^, ^55^, ^56^ could enable further miniaturization of the driving electronics for integrated actuation, sensing, and control of arrays of modular HASELs.

The modular units of HASEL actuators developed in this work provide a framework to create soft robots with scalable features and tunable actuation characteristics. However, Figures S8b and S17c (Supporting Information) suggest that mechanical losses between adjacent actuators within the stack increase as the number of actuators within the stack increases, which ultimately inhibits the amount of mechanical work that is performed by the device. More effective methods of stacking actuators can be implemented to maintain efficient operation by incorporating more robust mechanical connections between actuators using permanent adhesives or a more sophisticated heat‐sealing technique that directly bonds adjacent actuators within the stack. Additionally, if the modular units require a restoring force greater than that of the elastomeric wrap or the load that the muscles are pushing on, one could employ techniques utilized by the mammalian musculoskeletal system. HASEL actuators could be grouped as antagonistic muscle pairs and utilize a more advanced polymeric wrap that acts as a connective and structural component like fascia. While the mammalian musculoskeletal system is designed for actuators that contract, this artificial system would be designed for actuators that expand.

The introduced fabrication technique is effective for first prototyping different designs of HASEL actuators and then rapidly manufacturing modular units of actuators once a design is considered suitable for a desired application. The easy‐to‐implement toolkit provides a wide audience of roboticists, researchers from neighboring fields, and even hobbyists with simple yet effective methods to explore, create, and power HASEL actuators with muscle‐like performance.

## 4 Experimental Section

*Heat Sealing the Dielectric Shell*: The dielectric shell of the HASEL actuators was made from biaxially oriented polypropylene (BOPP) (70 gauge, 5020 Polypropylene, Multi·Plastics) film with a thickness of 18 µm. From the manufacturer, both sides of the film are integrated with a heat‐sealable copolymer layer and one side of the film is corona‐treated for ink adhesion which makes this side hydrophilic. All dielectric shells were heat sealed using the fabrication process illustrated in Figure 2 and shown in Movie S1 (Supporting Information). A commercially available 3D printer (RepRap3D, MagicD A2) was repurposed as a CNC heat sealing device. The designs for the dielectric shell were initially drawn using computer‐aided design (CAD) software (SolidWorks 2017–2018), exported as a 2D drawing in a .dxf file, and then converted to G‐code using an open source software called “dxf2gcode.” The G‐code defined the path of the extruder tip of the CNC machine, which was heated to 195 °C and moved at a constant speed of 800 mm min^−1^. A 0.32 cm thick neoprene rubber sheet and Kapton film was placed on the base of the heat sealing device to help evenly distributed the force of the heat sealing tip. Two sheets of BOPP were sandwiched between two sheets of Kapton (TapeCase 1 mil, Dupont) and placed on the base plate of the CNC heat sealing machine (Figure 2a). Kapton was used to evenly distribute the heat during the sealing process and to avoid melting through the BOPP. A thin film of Envirotemp FR3 was applied to the top surface of the Kapton to lubricate the extruder tip as it traveled across the heat sealing platform. Importantly, no FR3 was placed between the two layers of BOPP during the heat sealing process, as this compromised the strength of the seal. Figure 2b shows two different designs for the shell (a quadrant donut and a contracting HASEL) after they have been heat sealed. All heat seal designs included a small opening (2 mm wide) to act as a fill port.

The shape of the dielectric shell for donut HASELs was a circle with 50 mm diameter. A 2 mm gap in the perimeter of the circle served as a fill port for the dielectric liquid. For dimpled donut HASELs, the central heat seal was 1 mm in diameter and concentric with the 50 mm outer circle. For quadrant donut HASEL shells, the 50 mm circular heat seal was separated into four equal‐sized pouches each with their own fill port. The dielectric shell of curling and twisting HASEL actuators included a reservoir, shaped like a quarter of a circle, that was connected to a corrugated‐shaped heat seal. The contracting and curling HASELs were designed as seen in Figure 3b and Figure S2 (Supporting Information). The corrugated heat seal for twisting HASELs was designed as a Fibonacci spiral (Figure 3e). The dielectric shell for each of the actuators in the stack of folded actuators (Figure 9) is 30 mm × 30 mm.

*Filling the Shell with Liquid Dielectric*: A biodegradable vegetable‐based transformer oil (Envirotemp FR3, Cargill) was used as the liquid dielectric. A syringe with a needle was used to fill the chamber(s) of the heat sealed dielectric shell with the liquid dielectric through this port, Figure 2c. Air bubbles from the filling process were carefully removed. Then, a Kapton sheet was placed on top of the filled shell, and the fill port was sealed with a soldering iron heated to 195 °C, Figure 2d. Once sealed, the excess BOPP was cut away with scissors.

Dimpled donut HASELs were filled with 2 mL of FR3, while each of the four pouches of the quadrant donut HASELs was filled with 0.5 mL of FR3. The contracting, curling, and twisting HASELs were each filled with 5 mL of Envirotemp FR3. Each actuator in the stack of folded actuators (Figure 9) was filled with 0.76 mL of Envirotemp FR3.

*Electrodes for Actuators*: Polyacrylamide (PAAm) hydrogels swollen with lithium chloride (LiCl) solution were used and fabricated following the procedures developed by Bai et al.^57^ The hydrogel consists of aqueous solution of lithium chloride (LiCl; The Science Company, NC‐48518M) as the ionic conductor, Acrylamide (AAm; Sigma, A88887) as the monomer, *N*,*N*‐methylenebisacrylamide (MBAA; Sigma, 146072) solution as the cross‐linker, ammonium persulfate (AP; Sigma, 248614) as the ultraviolet (UV) light sensitive photo‐initiator, and *N*,*N*,*N*′,*N*′‐tetramethylethylenediamine (TEMED; Sigma, T9281) as the crosslinking accelerator. For a 10 mL solution of PAAm hydrogel, the following amounts were used: 9 mL of 8 mol L^−1^ LiCl, 1 mL of 0.933 g L^−1^ MBAA, 1.56 g of AAm, 0.03 g of AP, and 1–2 drops of TEMED. These hydrogels were cast into a 14 cm × 26 cm sheet on a film of BOPP which acted as a substrate making it easier to handle the gels, Figure S1 (Supporting Information). The BOPP sheet was placed (hydrophilic side up) onto a piece of glass (Figure S1a, Supporting Information). A 180 µm spacer made from polyethylene terephthalate (PET; School Smart, 631812) was used to control the thickness of the gels. The hydrogel solution was poured onto the BOPP (Figure S1b, Supporting Information) and another sheet of glass was placed on top of the spacer. Hydrogels were cured under a 365 nm UV light (Analytik Jena, XX‐40) for 1 h (Figure S1c, Supporting Information). Once cured, a laser cutter (Legend 36 EXT, Epilog) was used to cut electrodes from the sheet of BOPP‐backed hydrogels (Figure S1d, Supporting Information). Electrodes were placed onto the dielectric shell of the actuators with hydrogel facing the shell. The inherent tackiness of the hydrogels allowed the gels to stick to the dielectric shell without additional adhesives.

Additionally, we use an off‐the‐shelf, carbon‐based conductive paint (16056 DAG‐T‐502 Carbon Paint, Ted Pella, Inc.) as electrodes for actuators. Strips of copper tape were applied to opposite sides of the dielectric shell to act as electrical connections, and then the paint was brushed onto the actuator shell in the desired shape of the electrode. No modifications were made to this paint before application. A thin line of paint was applied to connect the electrode to the copper tape (Movie S1, Supporting Information).

For screen printed electrodes, a polyester screen with a mesh of 200 threads/cm (Gold‐Up USA) was used. A blank screen was coated on both sides with a thin layer of UV‐curable emulsion (Proclaim Dual Cure, Ulano). The emulsion dried on the screen in a light‐proof cabinet for a minimum of 4 h. A negative of the electrode pattern was printed in black ink onto a transparency film. Once the emulsion dried, the negative of the electrode pattern was placed on the screen and the screen was exposed to 365 nm UV light (Analytik Jena, XX‐40) for 10 min at a distance of 100 mm. The portion of screen covered by the electrode pattern remained uncured and was washed out of the screen with tap water. The screen was mounted to a flat surface with a pair of hinges to maintain position and a layer of electrodes was screen‐printed onto a glass plate to act as a guide. Sheets of heat sealed BOPP were placed onto the glass plate and the screen was flooded with conductive carbon ink (CI‐2051, Engineered Materials Systems, Inc), as shown in Figure S17a (Supporting Information). An 85a durometer squeegee (Gold‐Up USA) was pulled across the screen with constant pressure and speed to deposit the ink through the screen and onto the BOPP (Figure S17b, Supporting Information). The screen used was 304.8 mm × 609.6 mm which was large enough to accommodate two stacks of actuators, each consisting of eight individual actuators with 15 mm × 30 mm electrodes (Figure S17c,d, Supporting Information).

The electrodes for all donut HASEL actuators were 30 mm in diameter and concentric to the outer 50 mm circular heat seal. The electrodes for the contracting, curling, and twisting HASELs were the same shape and size as the reservoir of liquid dielectric. The strain limiting layer used for the curling and twisting actuators was a 115 µm thick laminating film (Heavy Weight Laminating Film, Grafix) that had an adhesive backing on one side. This laminating film was cut in the shape of the corrugated pathway and placed on one side of the actuator with the adhesive side in contact with the shell.

*Test Methods*: The dynamic response and equilibrium displacement of all actuators were recorded either directly with a laser displacement sensor (Model LK‐H057, Keyence) (Figure 4d–f and Figure S6, Supporting Information), or with a high‐speed camera (Model Phantom v710, Vision Research) and processed by Tracker video analysis and modeling software (version 4.96) (Figure 4c,g–h and Figure S5, Supporting Information). All actuators for performance tests were powered by a Trek high‐voltage power amplifier (Model 50/12). An NI DAQ (Model USB6212) was used to control the Trek based on custom LabVIEW programs (version 15.0.1f2), which were used to generate the waveforms seen in Figure S10 (Supporting Information) and trigger the high‐speed camera for frequency experiments. A stainless‐steel rod, constrained by a linear ball bearing, was used to apply vertical loads to the donut HASEL actuators (Figure S6, Supporting Information). For the frequency response measurements an acrylic frame with a spring‐loaded acrylic plate was used to provide a restoring force to the top of the actuators (Figure 4g). Orientation tests of the more details on the calculations of performance metrics can be found in the Supporting Information.

*Electronics*: The portable HV electronics were powered by a 3.7 V, 500 mAh lithium ion polymer battery (Li‐Polymer 503035, Adafruit) with a PowerBoost 1000 (2030, Adafruit), which amplified the output to 5 V. This 5 V output was used to supply power to the high‐voltage amplifier (Series 5VV10, Pico Electronics), HV switching circuit consisting of four optocouplers (OC100HG, Voltage Multipliers Inc.) oriented in an H‐bridge, and microcontroller (Longrunner Mini Nano V3.0). A detailed list of components used to construct the portable HV electronics can be seen in Table S1 (Supporting Information). More details about the portable HV circuit can be found in the Supporting Information.

## Conflict of Interest

S.K.M., E.A., N.K., and C.K. are listed as inventors on a U.S. provisional patent application (62/813266) and PCT applications (PCT/US2018/023797 and PCT/US19/020568) which cover fundamentals and basic designs of HASEL actuators as well as methods of fabricating and stacking actuators. S.K.M., E.A., N.K., and C.K. are co‐founders of Artimus Robotics, a start‐up company commercializing HASEL actuators.

## Supporting information

Supplementary

Supplementary

Supplementary

Supplementary

Supplementary

Supplementary

Supplementary

Supplementary

Supplementary

Supplementary

Supplementary
