# Supplementary material for: An Easy‐to‐Implement Toolkit to Create Versatile and High‐Performance HASEL Actuators for Untethered Soft Robots
Source: Adv Sci (Weinh). 2019 Jun 11;6(14):1900178. doi: 10.1002/advs.201900178 (PMC6662077; doi:10.1002/advs.201900178)
Supplement: Supplementary file 1 — Supplementary [file ADVS-6-1900178-s002.pdf]

Copyright WILEY-VCH Verlag GmbH & Co. KGaA, 69469 Weinheim, Germany, 2019.

ADVANCED SCIENCE Open Access

Supporting Information

for *Adv. Sci.,* DOI: 10.1002/advs.201900178

An Easy-to-Implement Toolkit to Create Versatile and High-Performance HASEL Actuators for Untethered Soft Robots

*Shane K. Mitchell, Xingrui Wang, Eric Acome, Trent Martin, Khoi Ly, Nicholas Kellaris, Vidyacharan Gopaluni Venkata, and Christoph Keplinger**

Copyright WILEY-VCH Verlag GmbH & Co. KGaA, 69469 Weinheim, Germany, 2019.

Supporting Information

Title: *An Easy-to-implement Toolkit to Create Versatile and High-performance HASEL Actuators for Untethered Soft Robots*

*Shane K. Mitchell, Xingrui Wang†, Eric Acome†, Trent Martin, Khoi Ly, Nicholas Kellaris, Vidyacharan Gopaluni Venkata, Christoph Keplinger**

Testing performance of actuators

Tip velocity and tip angular velocity of curling actuators. The dynamic performance of curling HASEL actuators was obtained using an applied voltage of 8 kV. The movement of the tip of the actuator was optically tracked by a high-speed camera (Model Phantom v710, Vision Research) with frame rate of 5,000 fps and was analyzed by Tracker video analysis and modeling software (version 4.96). The raw data was smoothed by a lowpass filter in MATLAB (version R2013b).

Blocking force of curling actuators. The blocking force of curling HASELs for different actuation voltages was measured using a load cell (Phidgets, CZL639HD) through a bridge interface (Phidgets, PhidgetsBridge 4-Input). The electrical signal of the load cell was calibrited using standard weights and fitted into a linear function in MATLAB (version R2013b). An acrylic mount was used to fix the load cell (Figure S3b).

Strain. All values for strain were calculated using the equation $s = \frac{(L_f - L_i)}{L_i}$, where s is strain, L_i is the initial length (or thickness) of the actuator, and L_f is the length (or thickness) of the device when actuated (L_f - L_i is referred to as the stroke of the actuator).

Strain rate. Strain rate, or the change in strain as a function of time was determined for a stack of three quadrant donut HASEL actuators. A stainless steel rod, constrained by a linear bearing, was used to apply vertical loads to the stack of actuators (Figure S6a). A square-wave actuation signal (polarity reversing every cycle, Figure S10a) was applied to produce rapid expansion of the actuators and the period of the signal was 1 s so that the actuators were able to settle to a steady-state position. Displacement was measured using a laser displacement sensor (Keyence, Model LK-H057). Sampling rate of the laser displacement sensor was set to 20 kHz and a 1,024-point moving average filter was applied to the data. The maximum strain rate during expansion of the actuators was measured for several loads and voltage amplitudes (Figure 4c).

Specific power and work density. Specific power, or power-to-mass, was measured for a stack of quadrant donut HASEL actuators lifting a mass, m. The same data used for strain rate was used to calculate specific power. The displacement data, $y(t)$, was recorded using a laser displacement sensor (Keyence, Model LK-H057) at 20 kHz with a 1,024-point moving average filter. Velocity, $v(t)$, and acceleration, $a(t)$, were then calculated using MATLAB. A Savitsky-Golay filter was applied to the displacement data, $y(t)$ to limit the amount of noise in the calculated velocity, $v(t)$, and acceleration, $a(t)$. With these kinematic parameters, power output, $P(t)$, of the quadrant donut HASEL actuators was calculated as the change in potential (PE) and kinetic energy (KE) of the mass, m with respect to time:

$$P(t) = \frac{\mathrm{d}(KE)}{\mathrm{d}t} + \frac{\mathrm{d}(PE)}{\mathrm{d}t} = \frac{\mathrm{d}(\frac{1}{2}mv(t)^2)}{\mathrm{d}t} + \frac{\mathrm{d}(mgy(t))}{\mathrm{d}t} = mv(t)[a(t) + g]. \quad \text{(S1)}$$

We report specific power or power output normalized to the mass of the actuator stack. Specific work during expansion was determined by integrating specific power from time t_1 to t_2. Average power was determined from the time, t_1, when the actuator began to expand, to the

time, t_2, when the actuator was no longer providing positive power to lift the load (Figure S6b-e). Average specific power was calculated as the specific work divided by t_2-t_1.

Frequency response. The dynamic response was determined for a stack of three quadrant donut HASELs by applying a voltage chirp signal with frequency ranging from 0.01 Hz to 1,000 Hz over 10 s. The polarity of the chirp signal was reversed every cycle (Figure S10c). Three springs, mounted in an acrylic frame (Figure 4g) were used to provide a restoring force to the stack of actuators. Actuation response was recorded using a high-speed camera (Model Phantom v710, Vision Research) with frame rate of 5,000 fps. Tracker video analysis and modeling software (version 4.96) was then used to determine the displacement. An NI DAQ (Model USB6212), controlled with LabVIEW software (version 15.0.1f2), was used to generate the chirp signal and trigger the high-speed camera. The magnitude and phase of the actuator response was estimated using the tfestimate function in MATLAB (version R2016a). A 350-sample Hann window was used to divide the signal into segments. The resulting Bode plot is shown in Figure 4 g,h.

Full-cycle electromechanical efficiency. The full-cycle electromechanical efficiency of quadrant donut HASEL actuators and foldable HASEL actuators was measured using the techniques and experimental setup shown in Figure S7. These measurements were based on the output work done by the actuator and the input electrical energy expended to the actuator during one cycle of operation. The cycle of operation was modeled after the work by Acome *et al.*,[23] which consists of four steps (Figure S7a):

1 – 2: A linear voltage ramp up to 8 kV was applied to the actuator. In response, the actuator lifted a 136-g-weight.

2 – 3: Once the actuator reached its maximum voltage and displacement, the load was removed using a mechanical solenoid as a brake.

3 – 4: The actuator was discharged down to 0 kV using a voltage ramp, which caused the device to return to its initial thickness. No mechanical work was performed during this step as the load was no longer applied to the actuator.

4 – 1: The 136-g-load was applied to the actuator at 0 kV. No electrical or mechanical work was done in this step.

The experimental setup seen in Figure S7b was used to measure the full-cycle electromechanical efficiency. A custom LabVIEW programs (version 15.0.1f2) and a National Instruments Data Aquisition Board (Model USB6212) were used to provide the voltage signals to the Trek HV amplifier (Model 50/12). The equivalent circuit for a HASEL actuator can be seen in Figure S7b, which consists of a variable capacitor, *C*, with a resistor in series, $R_{\text{electrode}}$, to represent resistance of the electrodes, and a resistor in parallel, R_{leakage}, to represent leakage current.

Electrical energy was determined by providing and monitoring a voltage ramp to the actuator, V(t), and measuring the current, I(t), while the actuators charged and discharged (Figure S7c and e). Current was calculated using Ohm's law with the measurement of a voltage, V_m(t), across a precision resistor, R_{m} = 2 kΩ. The precision resistor was placed on the ground side of the circuit and connected in parallel to a 470 nF capacitor, C_{m}, which acted as a low-pass filter. Charge, Q(t), was calculated by integrating the current over time, *t* (Figure S7e). Finally, electrical energy was calculated as the area enclosed by the path of the cycle within the voltage-charge work-conjugate plane (Figure S7g).

Mechanical energy was determined by measuring the displacement of the load as a function of time using the laser displacement sensor (Keyence, Model LK-H057), Figure S7d. The acceleration of the load as a function of time, a(t), was found by differentiating the displacement data twice, and then this information was used to calculate the force generated by the actuators as a function of time using $F(t) = \mathrm{m}[a(t) + \mathrm{g}]$, where m is the mass of the

load and *g* is the acceleration due to gravity, Figure S7f. The output mechanical energy is the area enclosed by a full-cycle in the force-displacement work-conjugate plane, Figure S7g.

Actuator performance at different orientations. The strain of a modular unit of quadrant donut HASELs as a function of voltage was conducted with the unit in three different orientations. The unit was adhered to an acrylic substrate using 3M 300LSE double sided tape. The stroke of the actuator was measured from the free end of the actuator using a laser displacement sensor (Keyence, Model LK-H057) and correlated to strain. Activation voltages were provided by the Trek (Model 50/12) using the reversing polarity signal seen in Figure S10b at 1 kV increments from 0 – 10 kV. The acrylic substrate was mounted in different orientations relative to the direction of gravity; the experiment was conducted with the actuator in an upright orientation (expanding upwards), sideways orientation (expanding horizontally), and upside-down orientation (expanding downwards).

Electronics

Portable HV electronics package. A schematic of the custom high voltage (HV) electronics package is shown in Figure S12a. The circuit was powered by a 3.7 V, 500 mAh lithium ion polymer battery (Li-Polymer 503035, Adafruit) with a PowerBoost 1000 (2030, Adafruit) which amplified the output from the battery to 5 V. The input to the HV amplifier ranged from 0-5 V and was regulated using a buck converter, which consisted of a MOSFET (Q2), a Schottky diode (D1), an inductor (L1), and a capacitor (C3). Output of the buck converter was varied using a potentiometer (ADJ_V). An Arduino program converted the analog signal from the potentiometer to the duty cycle of a PWM signal which was fed into the MOSFET of the buck converter.

An H-bridge constructed from four optocouplers (OC1, OC2, OC3, and OC4) was used for high-speed, HV switching. Only two optocouplers would be needed to switch HV on and off, however four optocouplers enabled us to also reverse polarity of the HV output. The current drawn by the infrared light emitting diodes (LEDs) of the optocouplers was limited using four resistors (R1, R2, R3, and R4). The HV outputs of the H-bridge, H1 and H2, are connected to the electrodes of the HASEL actuators. The H-bridge can be used to switch the polarity of the voltage applied to the actuators and discharge the actuators. Below is a description of each mode of operation for the H-bridge HV switching circuit.

First polarity: A 5 V signal was supplied to Q1 which activated OC1 and allowed HV charge to flow to H1. At the same time, a 0 V signal is supplied to Q3 which activated OC4 and connected H2 to ground.

Second polarity: A 0 V signal was supplied to Q1 which activated OC2 and connected H1 to ground. At the same time, a 5 V signal is supplied to 3 which activated OC3 and allowed HV charge to flow to H2.

Discharge: A 0 V signal is supplied to both Q1 and Q3 which activated OC2 and OC4, respectively, and connected both H1 and H2 to ground.

For periodic actuation signals, we switched polarity of the HV signal every cycle. This prevents charge retention in the HASEL actuators.

An Arduino code controlled the speed of switching, and therefore the frequency of actuation by reading an analog signal from a potentiometer (ADJ_F) and converting that signal to an analog input to the microcontroller. Figure S12b shows the finished printed circuit board (PCB). Table S1 provides a list of vendors and part numbers for each component of the HV circuit.

Multi-channel control of HASEL actuators using a joystick. For independently controlling multiple HASEL actuators, a three-channel HV power supply was constructed using three of

the electronics packages shown in Figure S13b. The three power supplies were connected as seen in Figure S13a. A microcontroller (Elegoo, EL-CB-001 UNO) using a custom Arduino code mapped analog signals from the joystick controller to each of the three power supplies. This mapping was developed by representing all possible locations of the joystick within the area of a unit circle, whose origin coincided with the origin of the Cartesian coordinate system, Figure S13b. The three actuators were represented as unit vectors $\overrightarrow{v_1}$, $\overrightarrow{v_2}$, and $\overrightarrow{v_3}$, on the unit circle, where each actuator was 120° apart, and $\overrightarrow{v_1}$ coincided with the positive x-axis. Similarly, the physical location of the joystick was represented as a vector, $\vec{u}$, (highlighted in red) with a length that varied from 0 to 1. When the joystick was at its origin, magnitude of the vector, $|\vec{u}|$, was zero. When the joystick was fully tilted, the magnitude of the vector $|\vec{u}|$ was 1. The magnitude of the voltage which was applied to any of the actuators depended on the location of the joystick in the unit circle. This voltage regulation was based on the projection of $\vec{u}$ onto the unit vectors of each actuator, where $\overrightarrow{p_1}$, $\overrightarrow{p_2}$, and $\overrightarrow{p_3}$ are the projections onto $\overrightarrow{v_1}$, $\overrightarrow{v_2}$, and $\overrightarrow{v_3}$, respectively. Voltage applied to the actuator was non-zero and scaled linearly by the magnitude of the projected vector if and only if the projected vector was pointing in the opposite direction of the unit vector of the actuator. An example is given in Figure S13b – when the joystick is moved to the first quadrant of the unit circle, the projected vectors $\overrightarrow{p_1}$ and $\overrightarrow{p_3}$ are in the same direction as $\overrightarrow{v_1}$ and $\overrightarrow{v_3}$, respectively, so the actuators associated with $\overrightarrow{v_1}$ and $\overrightarrow{v_3}$ do not activate. However, the projected vector $\overrightarrow{p_2}$ is in the opposite direction of $\overrightarrow{v_2}$, so the actuator described by the vector $\overrightarrow{v_2}$ activates, and the magnitude of the voltage applied to this actuator is proportional to $|\overrightarrow{p_2}|$. The magnitude of the voltage applied to each actuator was limited to 8 kV to prevent accidental electrostatic discharge across the PCB. It was observed that when the joystick was at rest at its origin (x = 0 and y = 0), the actuators would not always discharge appropriately. This issue was attributed to imperfections of the joystick, which prevented it from perfectly returning to its origin. To circumvent this

issue, a smaller circle concentric to the unit circle was constructed. Projected vectors with magnitude less than the radius of this second circle would cause the actuator(s) to discharge. Due to the need to reverse polarity between actuation cycles, this algorithm was designed so that the same polarity was never applied to the same actuator more than one time in a row. Additionally, the algorithm included a command which would activate all actuators simultaneously when the built-in switch of the joystick was pressed.

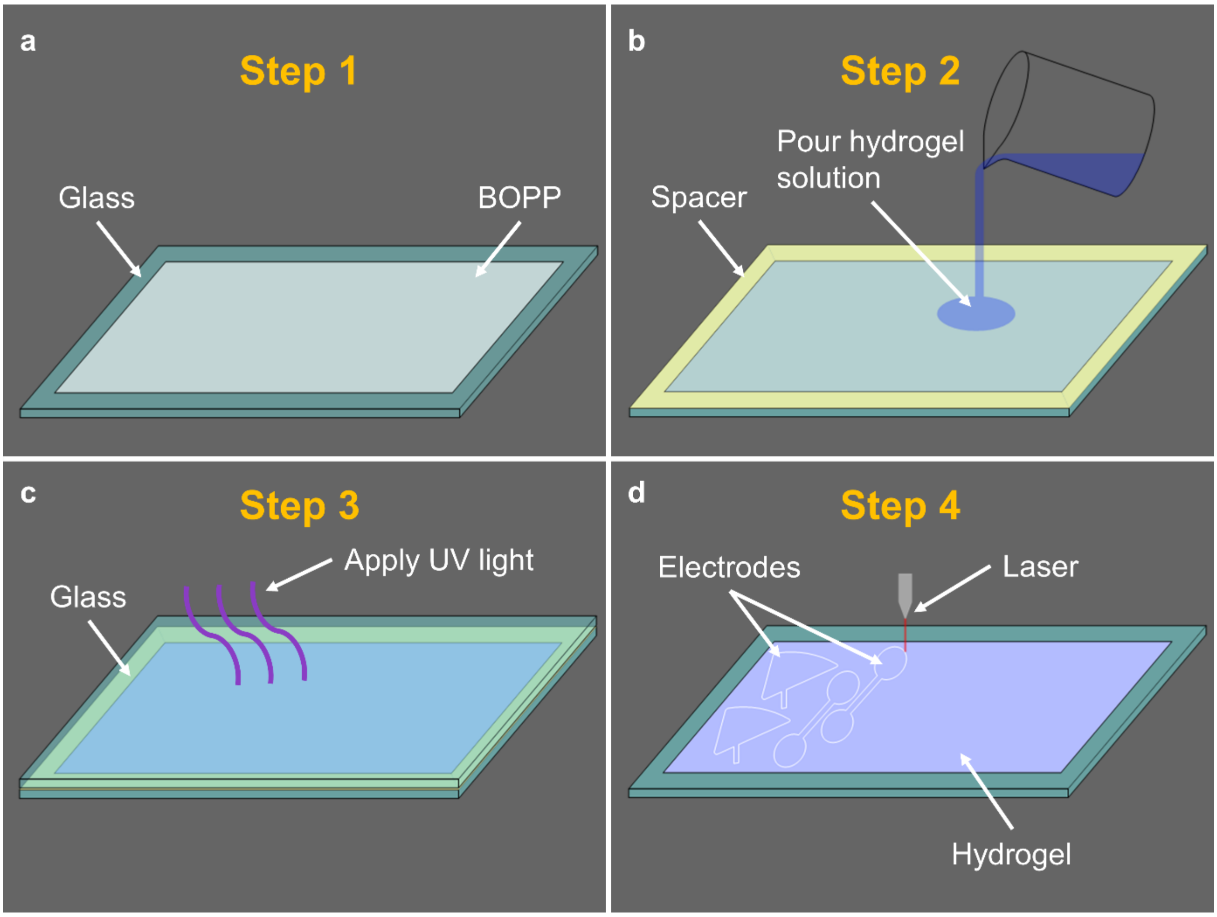

Figure S1. Fabrication process of hydrogel electrodes. a) A sheet of BOPP was placed on a piece of glass with its hydrophilic side up. b) A 180-µm thick PET spacer was placed on the glass around the perimeter of the BOPP. The hydrogel solution was poured onto the BOPP. c) Another piece of glass was placed on top of the hydrogel solution and the spacer. The hydrogel was exposed under 365-nm UV light for 1 hour to polymerize. d) Once polymerized, the top piece of glass and the spacer were removed, and the hydrogel (~160-µm-thick) with BOPP-backing was laser cut into the desired electrode shape.

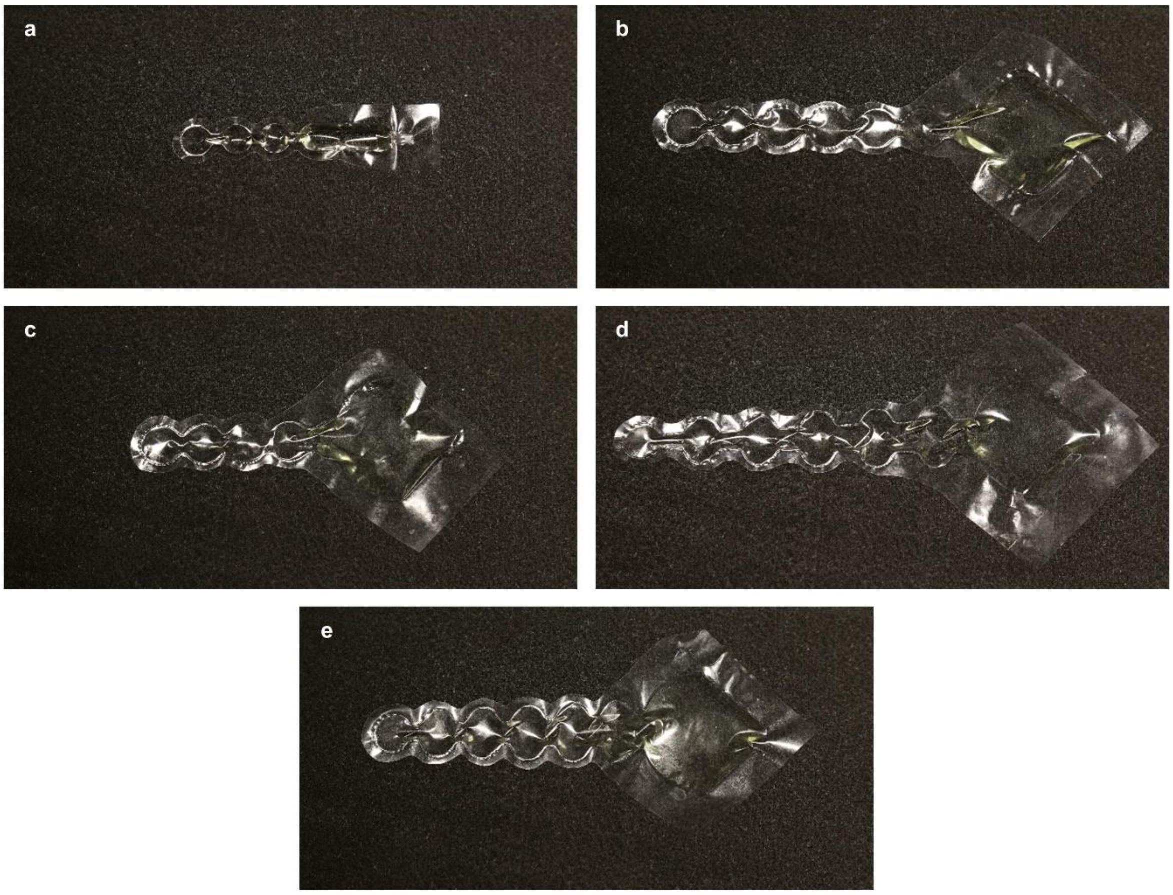

Figure S2. Five design iterations of contracting HASEL actuators.

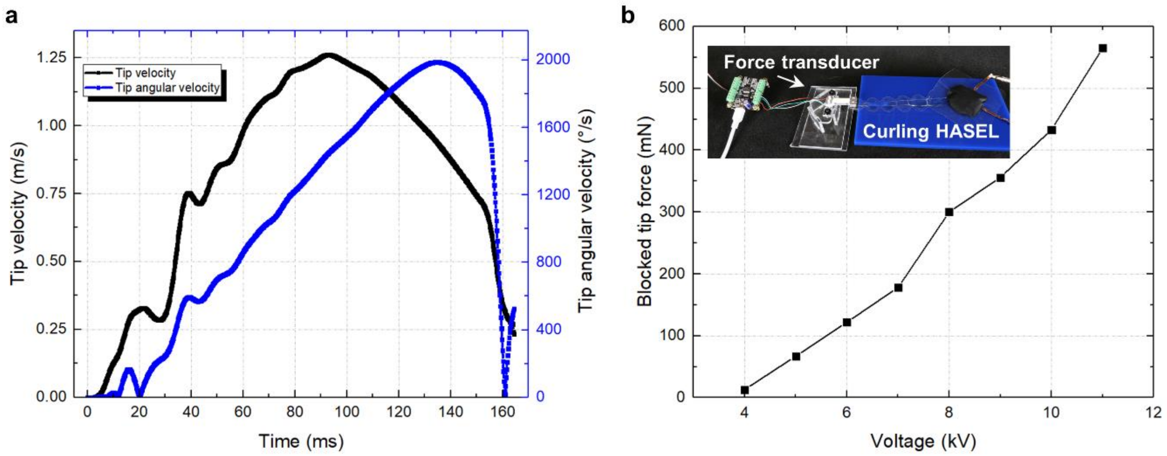

Figure S3. Performance metrics of curling HASEL actuators. a) The tip velocity in m s^{-1} and tip angular velocity in ^{o} s^{-1} was plotted against time. b) The blocked tip force in mN was plotted as a function of voltage.

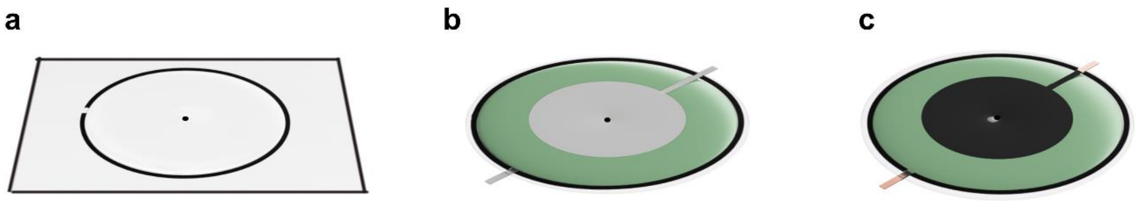

Figure S4. A schematic of a dimpled donut HASEL actuator. a) The dielectric shell of a dimpled donut HASEL actuator. b) A dimpled donut HASEL with ionic conductors (hydrogels) for electrodes and (c) electronic conductors (carbon-based paint) for electrodes.

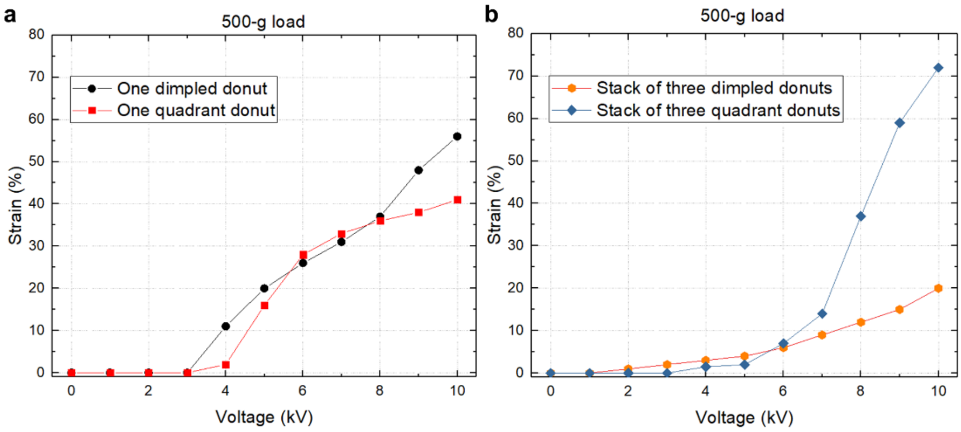

Figure S5. Comparison of dimpled donut and quadrant donut HASELs. a) Strain-voltage plot of one dimpled donut HASEL and one quadrant donut HASEL each under a 500-g load. b) Strain-voltage plot of a stack of three dimpled donut HASELs and a stack of three quadrant donut HASELs each under a 500-g load.

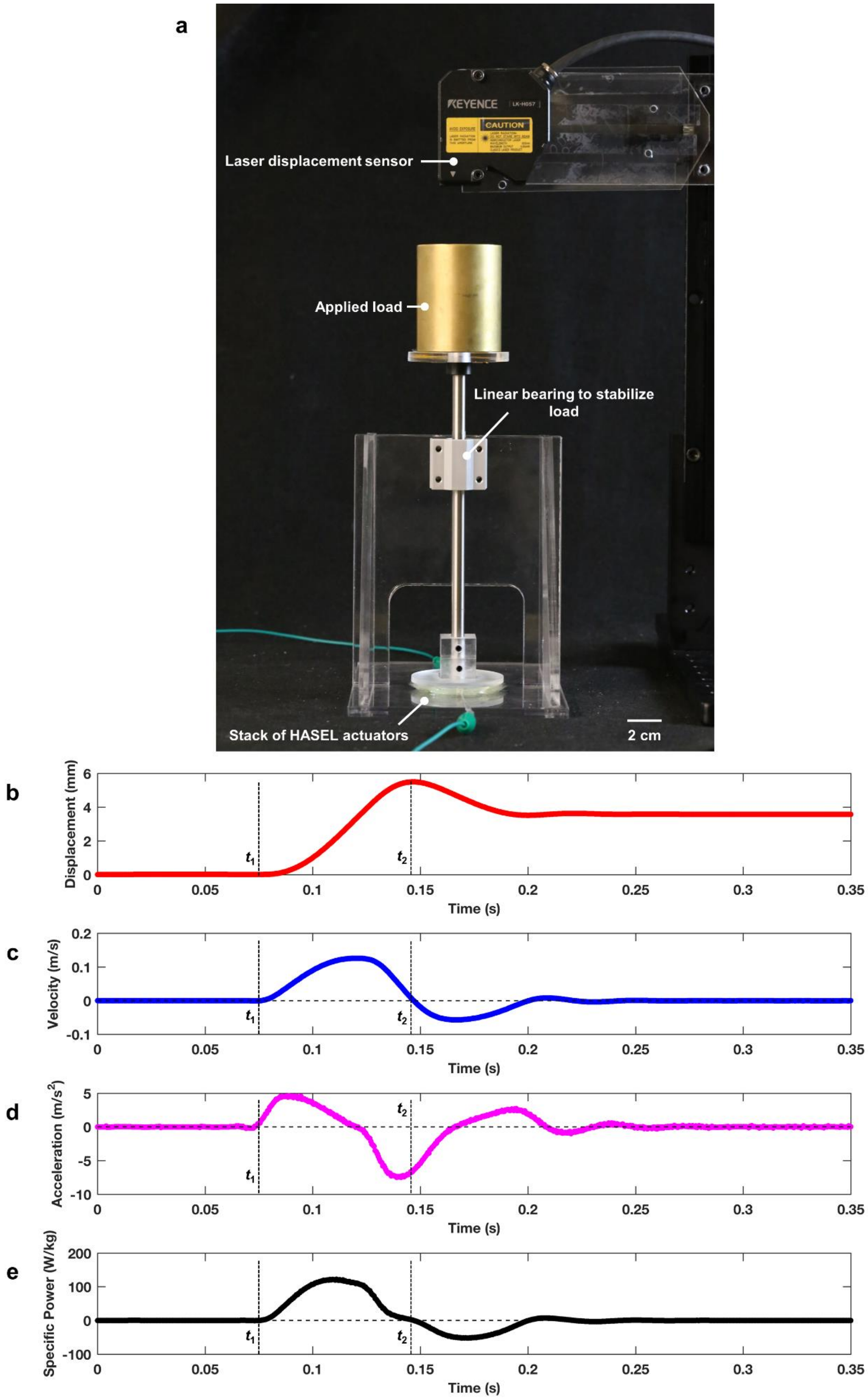
a
Laser displacement sensor
Applied load
Linear bearing to stabilize load
Stack of HASEL actuators
2 cm
b
Displacement (mm)
Time (s)
c
Velocity (m/s)
Time (s)
d
Acceleration (m/s^2)
Time (s)
e
Specific Power (W/kg)
Time (s)
t_1
t_2

Figure S6. Experimental setup and data acquired for calculation of specific power. a) Loads were applied directly to a stack of HASEL actuators through an 8 mm diameter stainless steel rod. A linear bearing limited the movement of the rod and load to only the vertical direction. Displacement of the load was recorded using a laser displacement sensor (Keyence, Model LK-H057). b) The recorded displacement was then used to calculate (c) velocity, and (d) acceleration of the load during actuation. e) Finally, these parameters were used to calculate specific power (Equation S1). Average power was determined from the time, t_1, when the actuator begins to expand, to the time, t_2, when the actuator is no longer providing positive power to lift the load. The data presented in plots (b-e) was collected from a stack of three quadrant donut HASELs operated at a 12-kV square wave signal at 0.5 Hz (Figure S6a) and under a 536-g load.

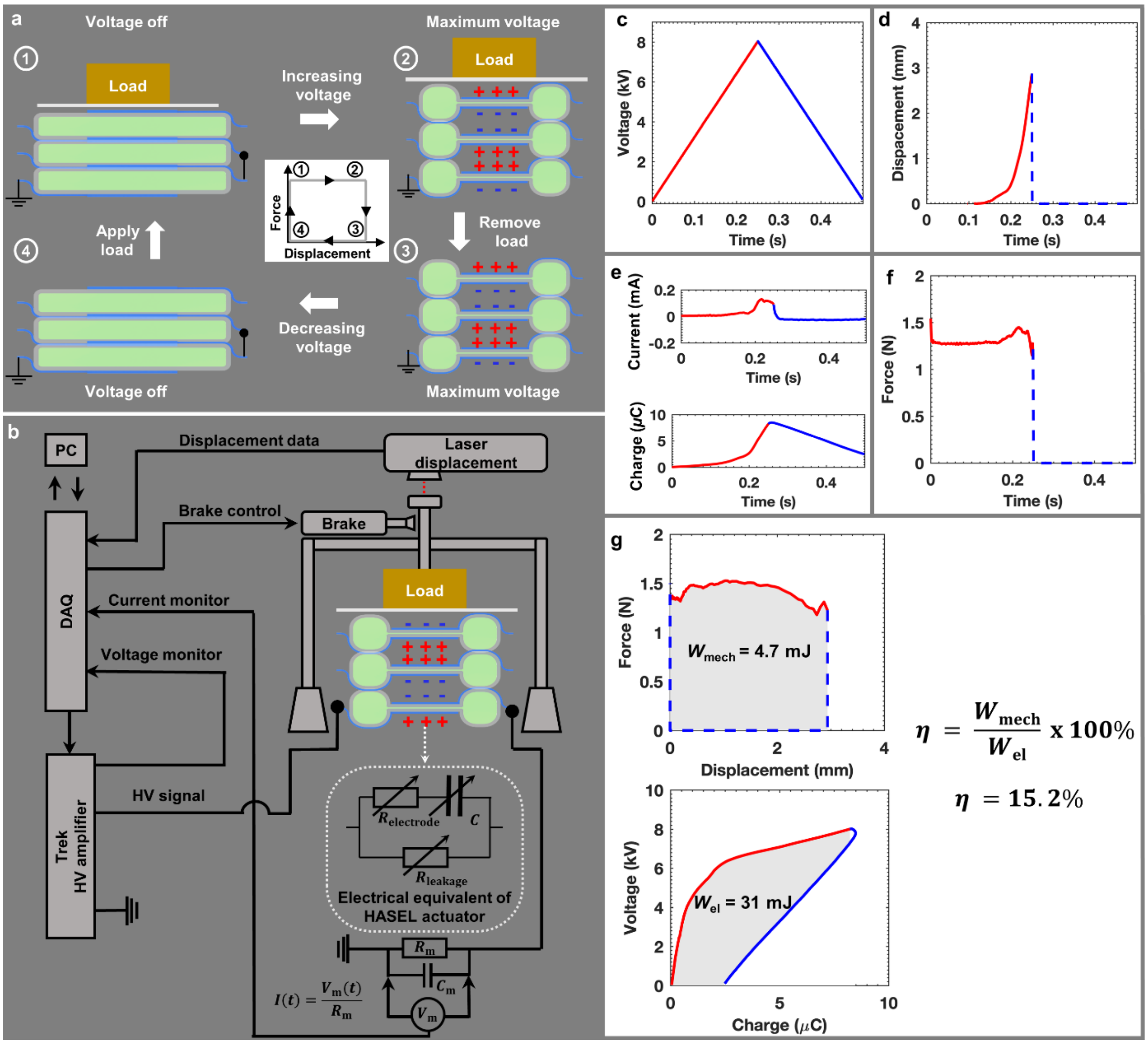

Figure S7. Experimental setup to measure the full-cycle electromechanical efficiency of HASELs. a) Experimental steps and cyclic path of the chosen experiment depicted in the force-displacement work-conjugate plane. The path traced by this cycle is approximately a rectangle for the relatively low mechanical loads used for these experiments. Thus, adding the load from points 4-1 does not noticeably alter the displacement state. From states 2-3, the load is fixed at its maximum displacement via the brake, while simultaneously the actuator starts to discharge, resulting in a vertical line on the force-displacement plot from 2-3. b) The experimental setup used to collect information about the electrical energy consumed by the actuators and the mechanical work done by the actuators. c-g) Experimental data collected for a stack of 3 quadrant donut HASELs under a 136-g-load. c) The applied voltage profile as a

function of time. d) The displacement of the actuator as a function of time. e) The current profile of the actuator as it charges and discharges. The current was integrated to obtain the amount of charge into and out of the actuator as a function of time. f) The force exerted by the actuator as a function of time. g) The force-displacement and voltage-charge work-conjugate planes were compared. The area enclosed by the cycle through the force-displacement plane represents the mechanical work achieved by the actuators and the area enclosed in the voltage-charge plane represents the amount of electrical energy consumed by the actuator. The work done by the actuator divided by the electrical energy consumed times 100% yielded the full-cycle electromechanical efficiency, 15.2% in this specific example.

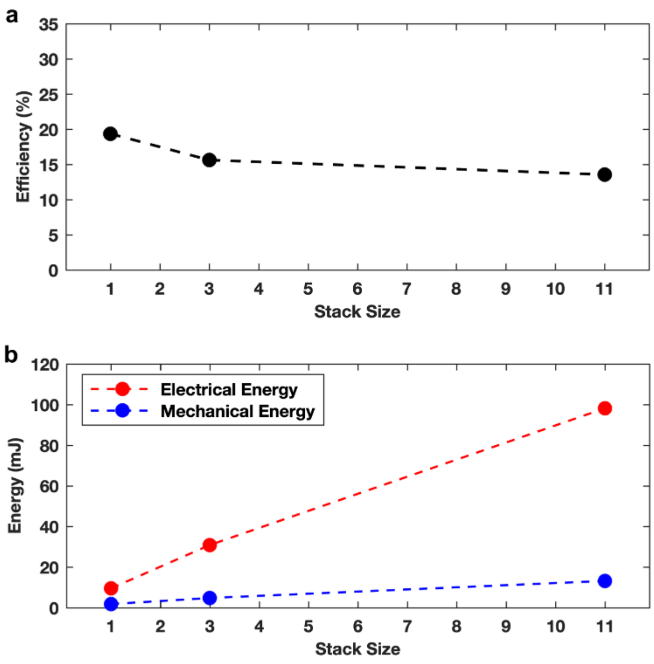

Figure S8. Full-cycle electromechanical efficiency of quadrant donut HASEL actuators as a function of the number of actuators in a stack. a) The efficiency of a single actuator, a stack of three actuators, and a stack of 11 actuators. b) A comparison of the electrical energy consumed and the mechanical energy output by the actuator(s) as the number of actuators in a stack was increased.

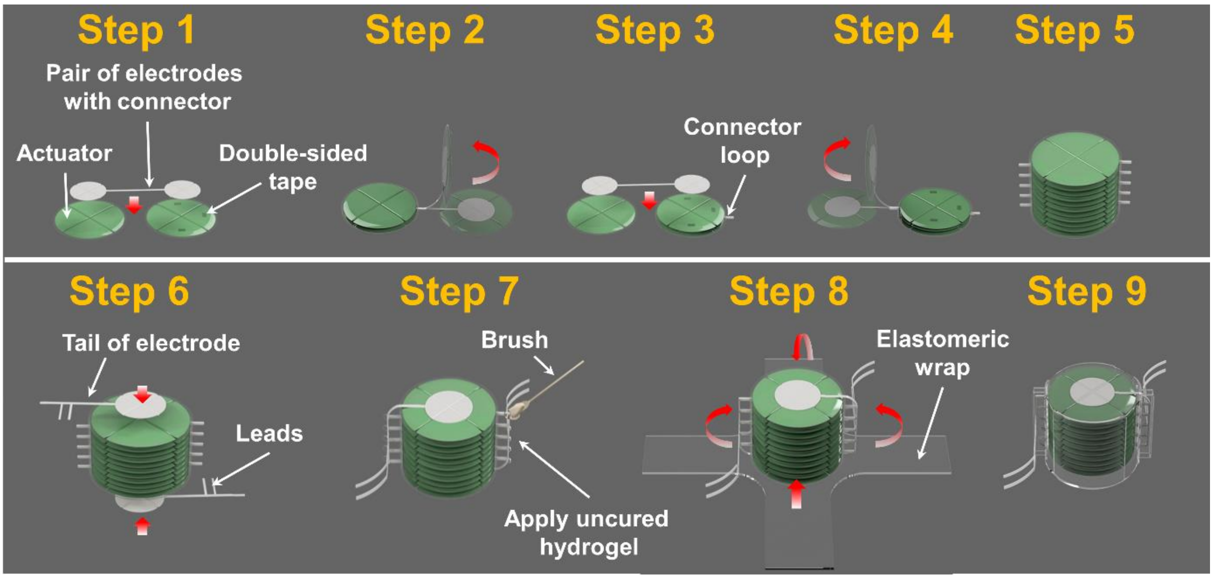

Figure S9. Stacking process to create modular units of quadrant donut HASELs. Step 1 – Hydrogel electrodes were laser cut in pairs with a connector. One electrode of the first pair was applied to the first actuator and the other electrode of the first pair was applied to the second actuator. Three strips (each 1 mm x 3 mm) of permanent double-sided tape (Scotch, 3M) were placed on the shell of the second actuator in locations that remained in contact with the first actuator during operation. Step 2 – The second actuator was folded on top of the first actuator with their heat seals aligned forming a stack of two actuators. This process created a loop of hydrogel electrode (from the connector between the pair of electrodes) which protruded from the one side of the actuators. Step 3 – One electrode of a second pair of electrodes was applied to the exposed side of the second actuator and the other electrode was applied to a third actuator. Three strips of double side tape were placed on the exposed side of the second actuator. Step 4 – The third actuator was folded on top of the second actuator, thereby creating a stack of three actuators. The loop of hydrogel created from this process protruded from the other side of the stack relative to the first loop. Double-sided tape was placed on the exposed side of the third actuator. Step 5 –The previous steps were repeated for subsequent actuators added to the stack, until the desired number of actuators was achieved (11 actuators for modular units described in this paper). Step 6 – Once 11 actuators were

stacked, electrodes were placed on the top and bottom of the stack. Each of these electrodes had tails which were long enough to contact all the loops of hydrogel protruding from a side of the stack. Additionally, the tails had two perpendicular leads which allowed electrical connection to a HV power supply, as well as electrical connection to adjacent modular units (Figure S14). Step 7 – Several drops of uncured hydrogel were applied between the electrode loops and the tails of the top and bottom electrodes, and the stack was exposed to 365-nm UV light for 1 hour or until the drops of hydrogel had polymerized. This process ensured that the electrodes of adjacent actuators were electrically connected. Step 8 – A 100-µm-thick elastomeric wrap made from Ecoflex 00-10 was placed under the stack. The wrap had four tabs which were wrapped around the stack of actuators. Step 9 – The completed modular unit of actuators.

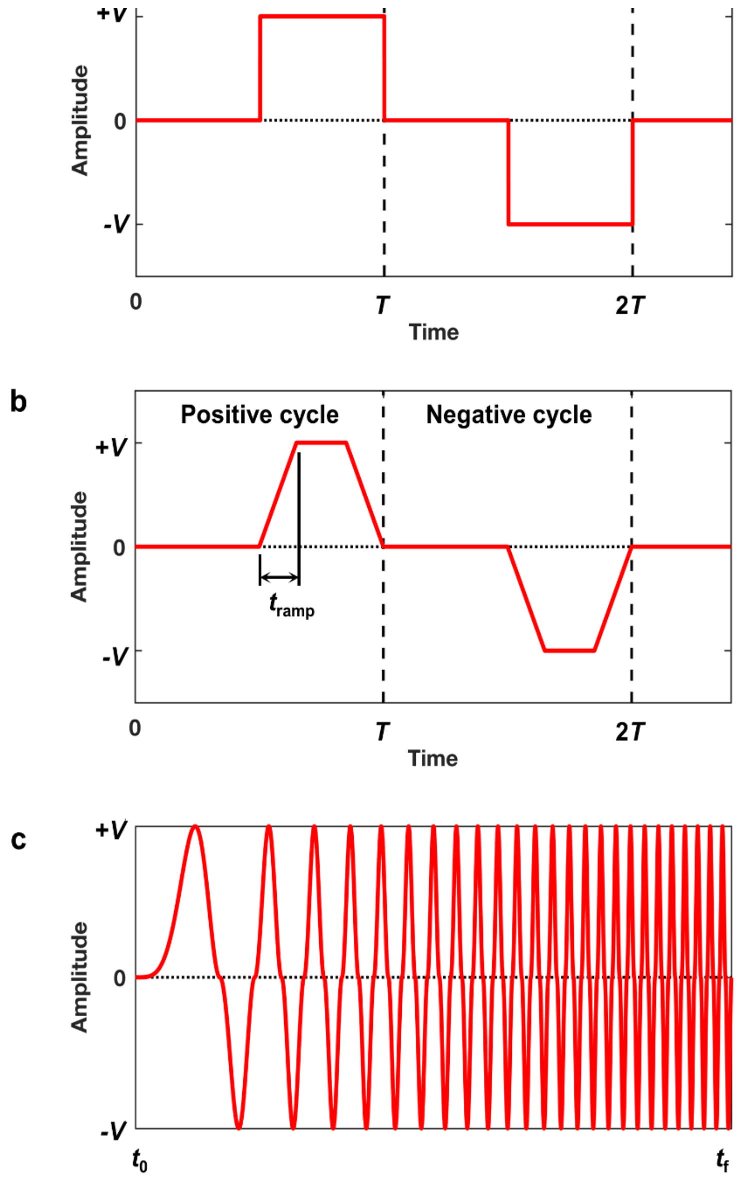

Figure S10. Voltage waveforms used for actuation and data collection. a) A square wave signal, with period *T*, that changes polarity every cycle switching between magnitude of +*V* and -*V*. b) A ramped square wave signal with ramp time of t_{ramp}. c) A chirp signal with frequency increasing linearly from t_0 to t_f.

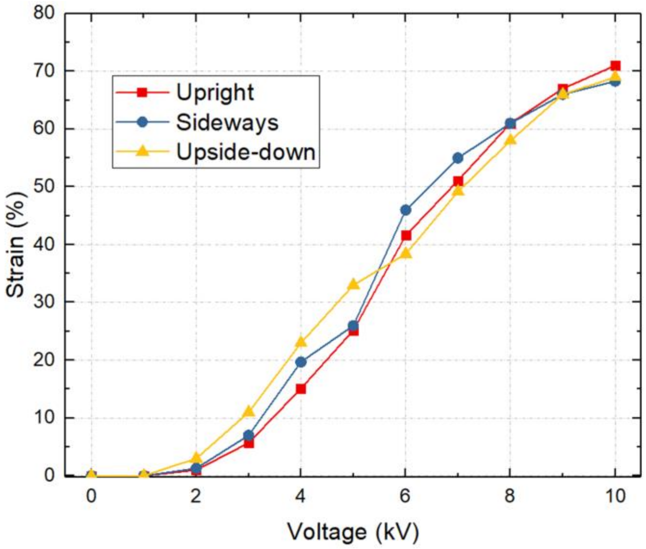

Figure S11. The effects of orientation relative to the direction of gravity on the actuation strain of a modular unit of quadrant donut HASELs. Actuation strain is plotted as a function of voltage for three different orientations (upright, sideways, and upside-down) of the actuator.

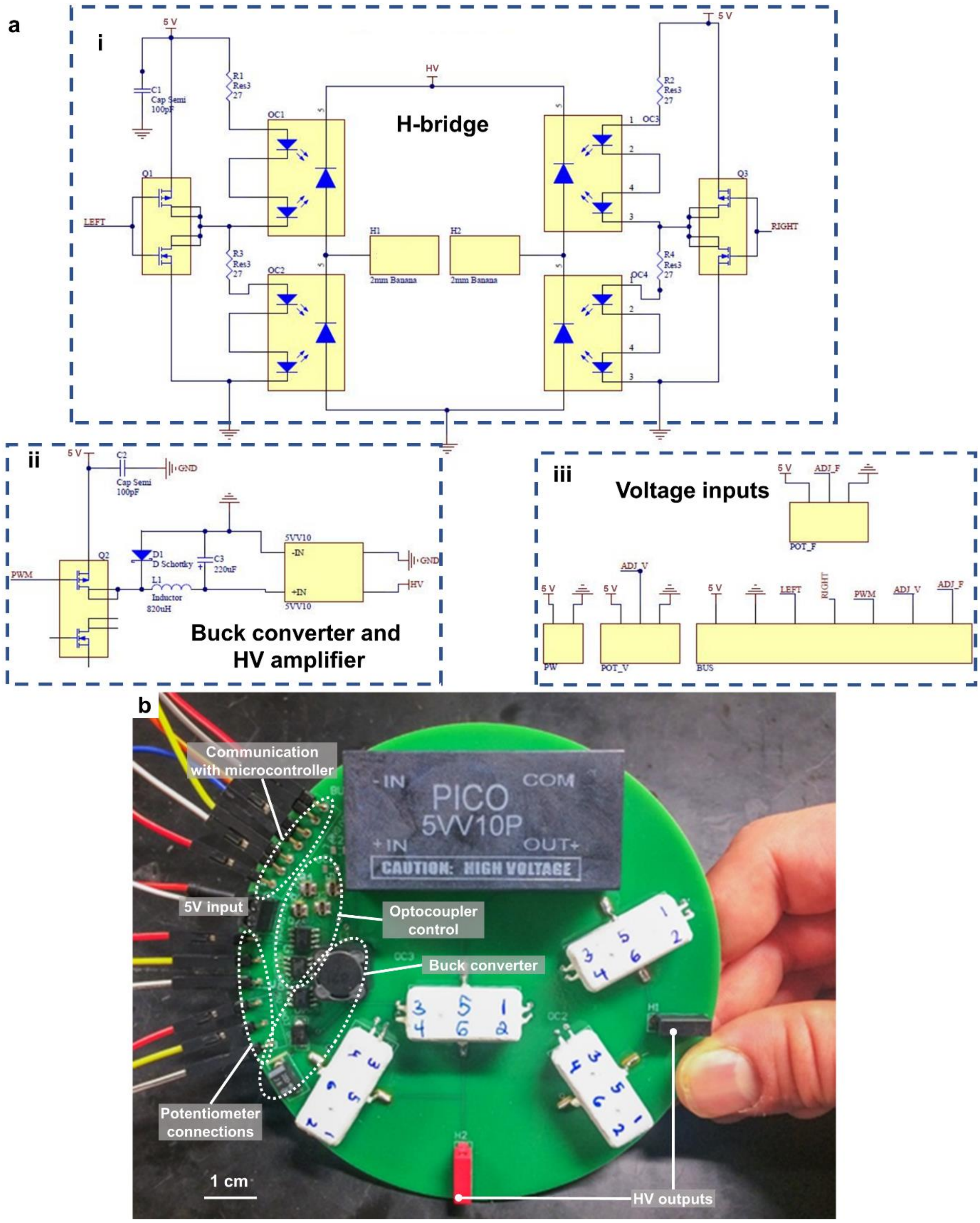

Figure S12. Portable high voltage power supply. a) The electrical schematic for the power supply which includes (i) an H-bridge constructed from optocouplers for HV switching, (ii) a buck converter to modulate the input voltage to the HV amplifier, and (iii) buses for voltage inputs. b) The completed power supply.

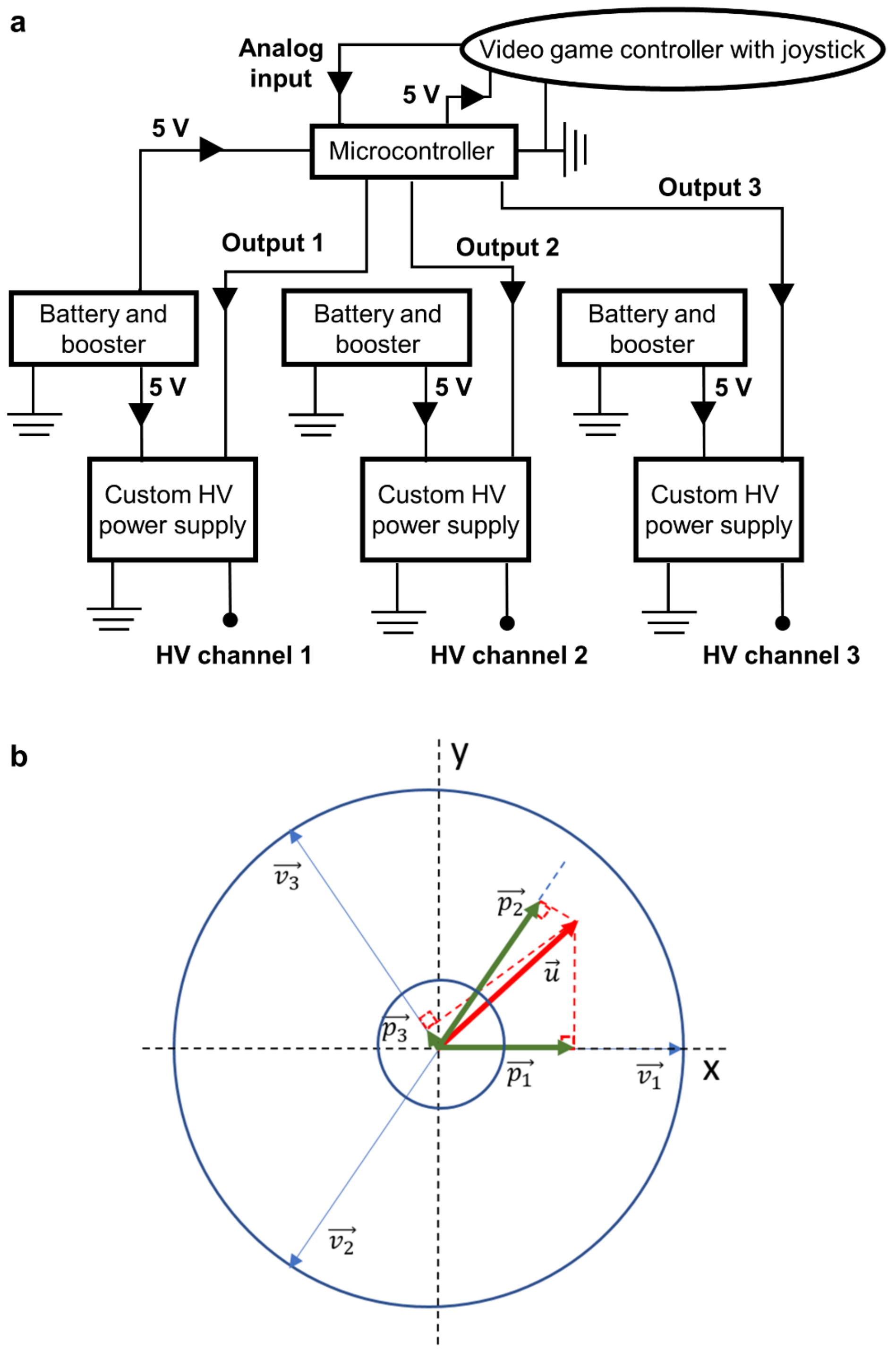

Figure S13. Three-channel high voltage power supply with joystick control. a) A block diagram of the three-channel power supply. The microcontroller supplied 5 V to the joystick controller. In turn, the controller sent analog signals to the microcontroller which then mapped those signals to each HV power supply. Outputs of the microcontroller sent a PWM signal to the buck converter of each power supply and a digital signal to the H-bridge of each power supply to control the optocouplers. Each power supply has its own battery and PowerBooster,

while one of these batteries also supplied power to the microcontroller. b) A unit circle which describes how the Arduino algorithm maps the position of the joystick to the outputs of the three-channel HV power supply.

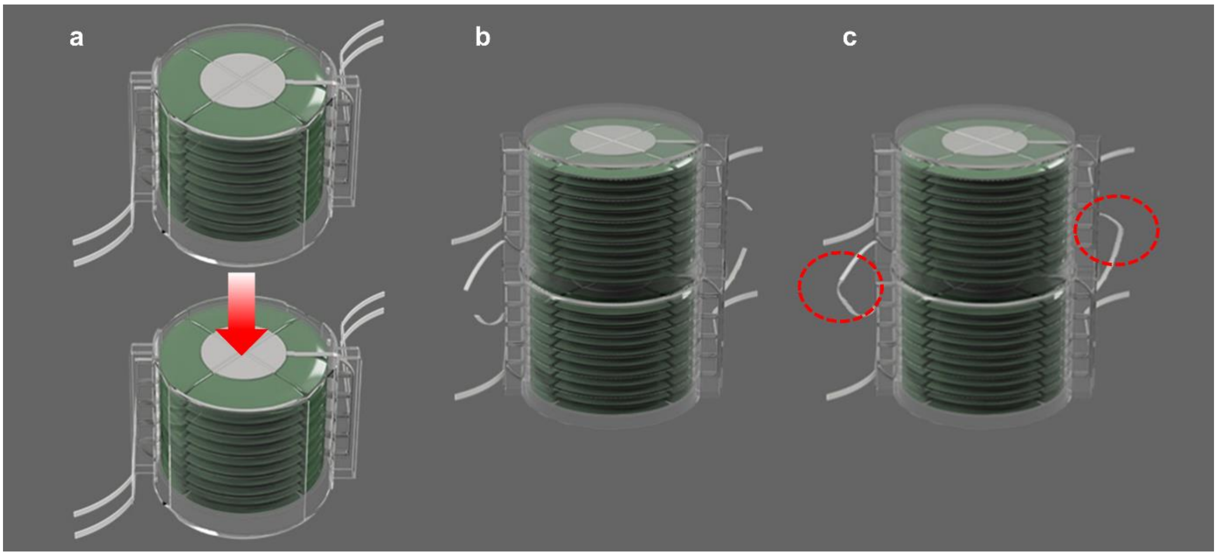

Figure S14. Stacking modular units of quadrant donut HASELs. a,b) Two modular units are stacked on top of each other. The elastomeric skin of each units provides adhesion between the modules. c) The top leads of bottom module are connected to the bottom leads of the top module, thereby electrically connecting the two modules.

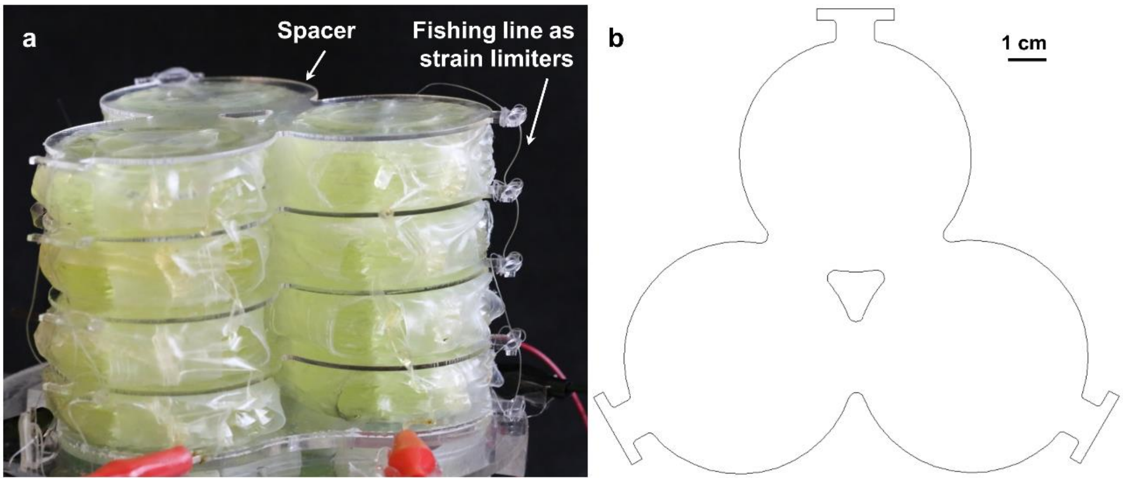

Figure S15. A soft continuum robot with fishing line as strain limiters and acrylic spacers. a) Fishing line was used to limit the strain of each modular unit to 100%. b) A sketch of the acrylic spacers used between each layer of three modular units.

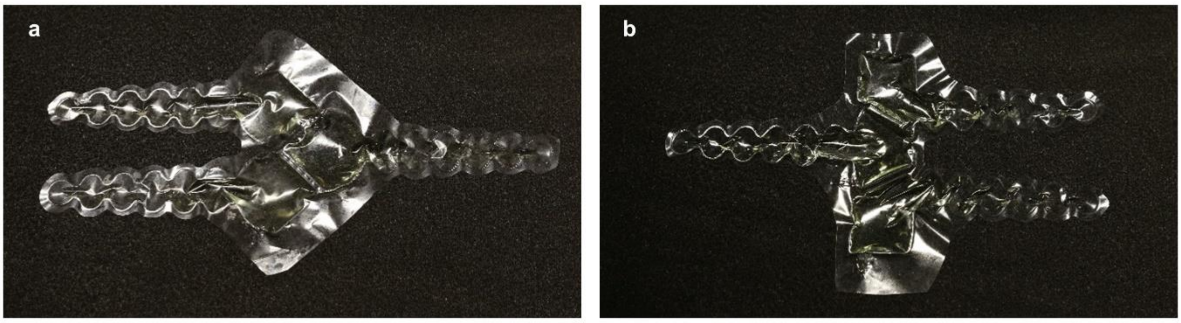

Figure S16. Two iterations of HASEL grippers based on curling HASEL actuators.

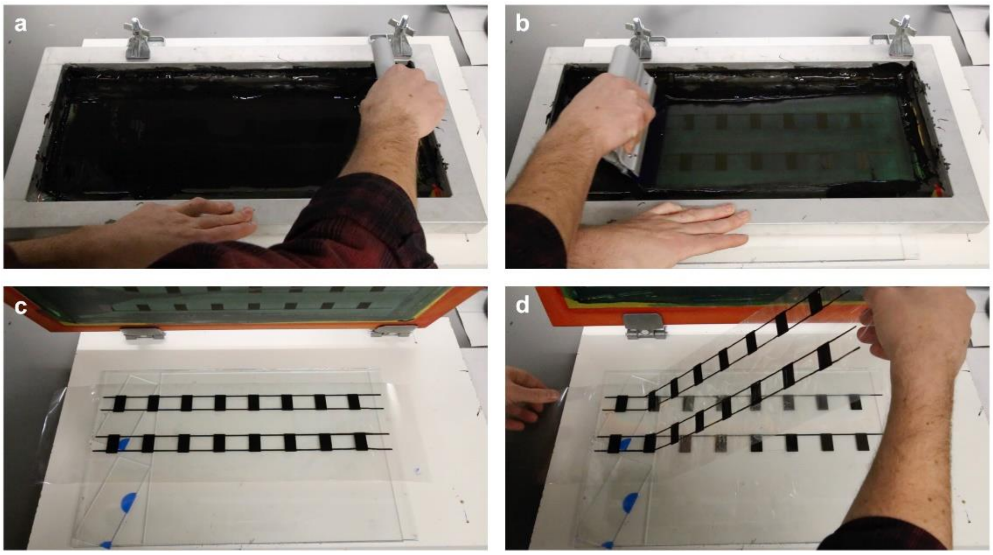

Figure S17. Screen printing conductive ink as electrodes. a) The heat-sealed BOPP sheet was placed underneath the screen and the screen was flooded with carbon-based conductive ink. b) Using a squeegee, the conductive ink was deposited through the screen onto the heat-sealed BOPP below. c) The screen is lifted off the substrate to reveal the electrodes. d) The sheet of BOPP is removed and left to dry before filling with liquid dielectric.

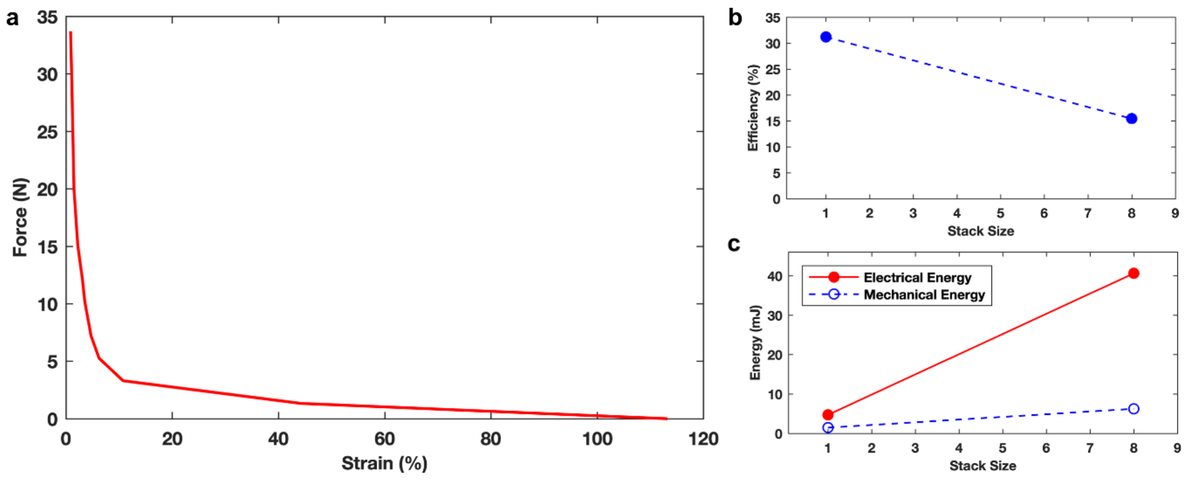

Figure S18. Performence metrics for foldable HASEL actuators. a) A force-strain plot for a stack of 8 folded HASEL actuators with an applied voltage of 8 kV. b) The efficiency of the folded actuators as a function of the number of actuators in the stack. c) The electrical energy consumed and the mechanical work done by the folded actuators as a function of the number of actuators in the stack.

Label	Description	Part Number
D1	Schottky diode	STMicroelectronics, STPS2L40U
L1	820 µH inductor	Bourns Inc., SRR1210A-821M
C1 & C2	100 pF capacitor	Murata Electronics GRM0335C1E101JA01D
C3	220 µF capacitor	Vishay, 293D227X9016E2TE3
R1, R2, R3, & R4	27 Ω resistor	Vishay, CRCW120627R0JNEAH P
Q1, Q2, & Q3	Dual MOSFET	ON Semiconductor, FDS8858CZ
OC1, OC2, OC3, & OC4	Optocouplers	Voltage Multipliers, OZ100SG

Table S1. List of components used for portable HV power supply shown in Figure S12.

Movie S1. Fabrication process to rapidly prototype HASEL actuators.

The fabrication process is separated into five key steps: **1)** print shell, **2)** fill with liquid dielectric, **3)** seal fill port, **4)** trim excess BOPP, and **5a)** apply hydrogel electrode or **5b)** paint carbon electrodes.

Movie S2. Curling HASEL actuators.

A curling HASEL actuator is used to mimic the strike of a scorpion tail and is shown to rapidly rupture a balloon upon activation. Three curling HASEL actuators are independently controlled with the three-channel high voltage power supply.

Movie S3. Twisting HASEL actuators.

A HASEL actuator is designed to resemble the shape of a Fibonacci spiral. Upon activation, the device simultaneously twists and curls.

Movie S4. Operating a modular unit of donut HASELs.

A stack of 11 quadrant donut HASEL actuators forms a modular unit and is shown actuating at 5 kV and 8 kV using the voltage waveforms in Figure S10a and b. The actuation frequency ranges from 0.25 Hz to 15 Hz. Additionally, the modular unit is shown to operate in a horizontal orientation.

Movie S5. High-power HASEL actuators that jump.

A stack of 11 quadrant donut HASELs is shown to jump at a voltage of 7.8 kV and frequency of 3.9 Hz in real-time and slow motion (0.0625x).

Movie S6. Portable electronics for untethered operation of HASELs.

A portable high voltage power supply is used to demonstrate untethered operation of a modular unit of HASEL actuators, with actuation voltage and frequency adjusted by two potentiometers. Additionally, the stack of HASEL actuators easily lifts the complete power supply, without tethers to additional sources of energy.

Movie S7. Soft robots with three-dimensional mobility powered by HASEL actuators.

Following the movement of a joystick controller, a soft continuum robot is shown to bend in multiple directions and to linearly expand.

Movie S8. A maze game operated by HASELs.

An interactive game is created by placing an acrylic maze on top of three modular units of HASEL actuators. A joystick is used to independently activate the modules, thereby tilting the maze in different directions to guide the marble through the correct path.

Movie S9. Lifting weights with HASELs.
Three columns of actuators each consisting of two modular units is shown to lift 1 kg at 40% strain when activated with 8 kV.

Movie S10. Towards a soft continuum robot: *Terry the trunk*
An untethered soft continuum robot is constructed using three columns each comprised of five modular units of HASEL actuators with an end effector made from three curling HASEL actuators. This continuum robot, named “Terry the trunk”, demonstrates the mobility and grasping capabilities to handle a number of delicate objects including a balloon, bag of chips, and a plastic cup.
